# Molecular principles of assembly, activation, and inhibition in epithelial sodium channel

**DOI:** 10.7554/eLife.59038

**Published:** 2020-07-30

**Authors:** Sigrid Noreng, Richard Posert, Arpita Bharadwaj, Alexandra Houser, Isabelle Baconguis

**Affiliations:** 1 Department of Biochemistry and Molecular Biology, Oregon Health & Science UniversityPortlandUnited States; 2 Vollum Institute, Oregon Health & Science UniversityPortlandUnited States; 3 Neuroscience Graduate Program, Oregon Health & Science UniversityPortlandUnited States; The University of Texas at AustinUnited States; University of British ColumbiaCanada

**Keywords:** heteromeric ion channel, cryo-electron microscopy, proteolysis, Human

## Abstract

The molecular bases of heteromeric assembly and link between Na^+^ self-inhibition and protease-sensitivity in epithelial sodium channels (ENaCs) are not fully understood. Previously, we demonstrated that ENaC subunits – α, β, and γ – assemble in a counterclockwise configuration when viewed from outside the cell with the protease-sensitive GRIP domains in the periphery (Noreng et al., 2018). Here we describe the structure of ENaC resolved by cryo-electron microscopy at 3 Å. We find that a combination of precise domain arrangement and complementary hydrogen bonding network defines the subunit arrangement. Furthermore, we determined that the α subunit has a primary functional module consisting of the finger and GRIP domains. The module is bifurcated by the α2 helix dividing two distinct regulatory sites: Na^+^ and the inhibitory peptide. Removal of the inhibitory peptide perturbs the Na^+^ site via the α2 helix highlighting the critical role of the α2 helix in regulating ENaC function.

## Introduction

The ability to balance the amount of water inside and outside cells is absolutely essential for life. In the specialized epithelial tissues, the apical expression of the epithelial sodium channel (ENaC) gives rise to a transepithelial directional flow of Na^+^ ions (Palmer and Frindt, 1986). ENaC function is therefore crucial in proper regulation of blood volume and pressure, as well as surface liquid volume in the respiratory and reproductive systems (Boggula et al., 2018; Hummler et al., 1996; Rossier, 2014). In humans, the essential role of ENaC in blood volume and pressure regulation is highlighted in gain-of-function mutations, as observed in Liddle syndrome, and also in loss-of-function mutations in pseudohypoaldosteronism type 1, severe genetic diseases that lead to hyper- and hypotension, respectively (Palmer and Alpern, 1998; Liddle and Coppage, 1963; Shimkets et al., 1994; Cheek and Perry, 1958; Hanukoglu, 1991; Strautnieks et al., 1996; Chang et al., 1996; Edelheit et al., 2005).

ENaC belongs to the ENaC/degenerin family, defined by Na^+^-selectivity, voltage independence, and amiloride sensitivity (Kellenberger and Schild, 2002). Members of this family, including the well-studied relative Acid-Sensing Ion Channel (ASIC), have subunits that consist of short intracellular N- and C-termini, two membrane-spanning helices, and a large cysteine-rich extracellular domain (ECD) that can form homo- or heterotrimeric ion channels (Jasti et al., 2007; Noreng et al., 2018). In the case of ENaC, three homologous subunits, α, β, and γ, form trimers which are arranged in a counterclockwise direction when viewed from the extracellular space (Noreng et al., 2018; Collier and Snyder, 2011; Collier et al., 2014; Chen et al., 2011). Seminal cloning and functional studies of the ENaC subunits demonstrated that while homomeric α and diheteromeric forms of ENaC containing α/β or α/γ can form functional ion channels, the α-β-γ presents robust Na^+^ currents indicating that the triheteromeric form is the favored assembly (Canessa et al., 1993; Canessa et al., 1994; Staruschenko et al., 2005; Lingueglia et al., 1993; Firsov et al., 1996).

Unlike other ion channels, ENaC activity is primarily modulated by proteases that remove peptidyl tracts in the ECD (Vallet et al., 1997; Sheng et al., 2006; Kashlan et al., 2015). Removal of these polypeptides irreversibly converts ENaC channels from a low-channel-activity state to constitutively active channels (Carattino et al., 2006; Bruns et al., 2007). Canonically, the α subunit is cleaved twice by furin, while the γ subunit is cleaved once by furin and once by prostasin (Carattino et al., 2006; Bruns et al., 2007; Hughey et al., 2004; Carattino et al., 2008a; Passero et al., 2010). Of note, the β subunit does not have canonical protease sites. Conversely, extracellular Na^+^ attenuates ENaC activity by binding to allosteric sites in the ECD, an effect referred to as Na^+^ self-inhibition (Fuchs et al., 1977; Awayda, 2016). Interestingly, cleavage of the α subunit has been shown to abrogate Na^+^ self-inhibition (Sheng et al., 2006). The molecular mechanisms of neither proteolytic activation nor Na^+^ self-inhibition are currently understood.

We have previously solved the first structure of human ENaC at a nominal resolution of 3.9 Å by cryo-electron microscopy (cryo-EM) (Noreng et al., 2018). The structure provided valuable insight into channel assembly, stoichiometry and positions of the protease-sensitive domains, deemed the **G**ating **R**elease of **I**nhibition by **P**roteolysis (GRIP) domain. This initial study took advantage of ENaC constructs biochemically designed to be resistant to endogenous proteases, trapping the molecule in the uncleaved state. Our structure showed critical structural divergence from close relative ASIC in the peripheral region of the ENaC ECD, particularly in the finger and the specialized GRIP domains, which are not found in ASIC (Jasti et al., 2007). Here, we determined the structure of ENaC by single-particle cryo-EM at 3 Å to gain molecular insight into the roles of Na^+^ and the protease-sensitive GRIP domains in ENaC function. The overall improvement of the map quality reveals for the first time the molecular source of the preferred channel assembly, and hints at mechanisms of Na^+^ self-inhibition and proteolytic activation.

## Results

### Determinants of channel composition

To investigate the structural source of ENaC trimer assembly, we exploited a set of constructs, deemed ENaC_FL_, which comprises wild-type α and β, and N-terminally eGFP-tagged γ, and behaves like wild-type ENaC as measured by electrophysiology (Figure 1—figure supplement 1 and Table 1). We solved a 3 Å cryo-EM structure of ENaC_FL_, based on the gold-standard Fourier shell correlation (Figure 1—figure supplements 2–4, Table 2). Resolution is higher in the channel core, calculated up to 2.6 Å, with β strands and smaller side chains clearly visible (Figure 1—figure supplements 4 and 5). To determine the structure of ENaC_FL_, we expressed ENaC_FL_ in HEK293T/17, solubilized in digitonin, and added two different Fabs, 7B1 (recognizes the α subunit) and 10D4 (recognizes the β subunit), to facilitate particle alignment (Figure 1—figure supplement 1b,c). Reference-free 2D class averages and 3D classifications reveal that ENaC_FL_ channels form as α-β-γ counterclockwise when viewed from outside the cell (Figure 1—figure supplements 2–4). However, the transmembrane domain (TMD) and the cytosolic domain (CD) were not resolved; we speculate that preferred particle orientation, air-water interface denaturation, and intrinsic protein flexibility and conformational heterogeneity contribute to the lack of 3D reconstruction of the TMD and CD (Figure 1—figure supplement 4f). Therefore, we did not include the TMD and CD portions in the ENaC_FL_ structure (Figure 1—figure supplement 4). The higher resolution of ENaC_FL_ structure affords us confidence in the placement of side chains for the first time, providing unprecedented insight into how the ECD mediates ENaC function.

**Table 1.** IC_50_ values of ENaC for three different blockers (amiloride, phenamil mesylate and benzamil). IC_50_ values (mean ± S.E.M) determined from dose-response curves for three different blockers (amiloride, phenamil mesylate and benzamil) at different holding voltages (-60 mV, -40 mV, -20 mV, 0 mV).

		IC50 values (nM)	
	Amiloride	Phenamil	Benzamil
0 mV -20 mV -40 mV -60 mV	97.14 ± 21.62 80.05* ±* 8.78 80.25 *±* 11.37 86.34 *±* 27.04	51.37 ± 10.42 49.97 ± 11.18 43.37 ± 11.86 51.01 ± 14.12	36.74 ± 13.25 29.41* ±* 6.47 27.72* ±* 6.65 32.90 ± 12.66

**Table 2.** Statistics of data collection, three-dimensional reconstruction, and model refinement.

		ENaC_FL_	
Pre-merge dataset	1	2	3
Material Source	Membrane	Whole cell	Whole cell
Detergent	Digitonin	Digitonin	Digitonin
Fab	7B1 and 10D4	7B1 and 10D4	7B1 and 10D4
Microscope	FEI Krios	FEI Krios	FEI Krios
Voltage (kV)	300	300	300
Detector	Gatan K2 Summit	Gatan K2 Summit	Gatan K2 Summit
Defocus range (*µ*m)	−0.8 – −2.2	−0.8 – −2.2	−0.8 – −2.2
Exposure time (s)	3	3	3
Dose rate (e*−*/Å^2^/frame)	1.0	1.0	1.0
Frames per movie	60	60	60
Pixel size (Å)	0.415	0.415	0.415
Total dose (e*−*/Å^2^)	60	60	60
Motion correction	UCSF MotionCor2	UCSF MotionCor2	UCSF MotionCor2
CTF estimation	CTFFIND 4	CTFFIND 4	CTFFIND 4
Particle picking	cryoSPARC blob	cryoSPARC blob	cryoSPARC blob
2D/3D classification	cryoSPARC 2.11	cryoSPARC 2.11	cryoSPARC 2.11
3D classification and refinement	Relion 3.0,	Relion 3.0,	Relion 3.0,
	cryoSPARC 2.11,	cryoSPARC 2.11,	cryoSPARC 2.11,
	cisTEM 1.0	cisTEM 1.0	cisTEM 1.0
Symmetry	C1	C1	C1
Particles processed	172 954	218 428	71 549
Resolution masked (Å)	3.57	3.05	3.96
Map Sharpening B-factor (Å^2^)	91.8	87.3	97.9
		cryoSPARC 2.11 merged map	
Merged Symmetry		C1	
Merged particle count		252 071	
Merged resolution masked (Å)		3.06	
		cisTEM 1.0.0 merged map	
Merged Symmetry		C1	
Merged particle count		248 079	
Merged resolution masked (Å)		3.11	
Initial model		6BQN	
Non-hydrogen atoms		11 740	
Protein residues		1 594	
Ligands (Na^+^, NAG)		1, 10	
Resolution (FSC = 0.143, Å)		3.06	
Molprobity score		1.37	
C*β* deviations		0	
Poor rotamers		0.84%	
Ramachandran outliers		0	
Ramachandran allowed		2.7%	
Ramachandran favored		97.3%	
Bond length rmsd (Å)		0.002	
Bond angle rmsd (°)		0.390	

**Figure 1.**
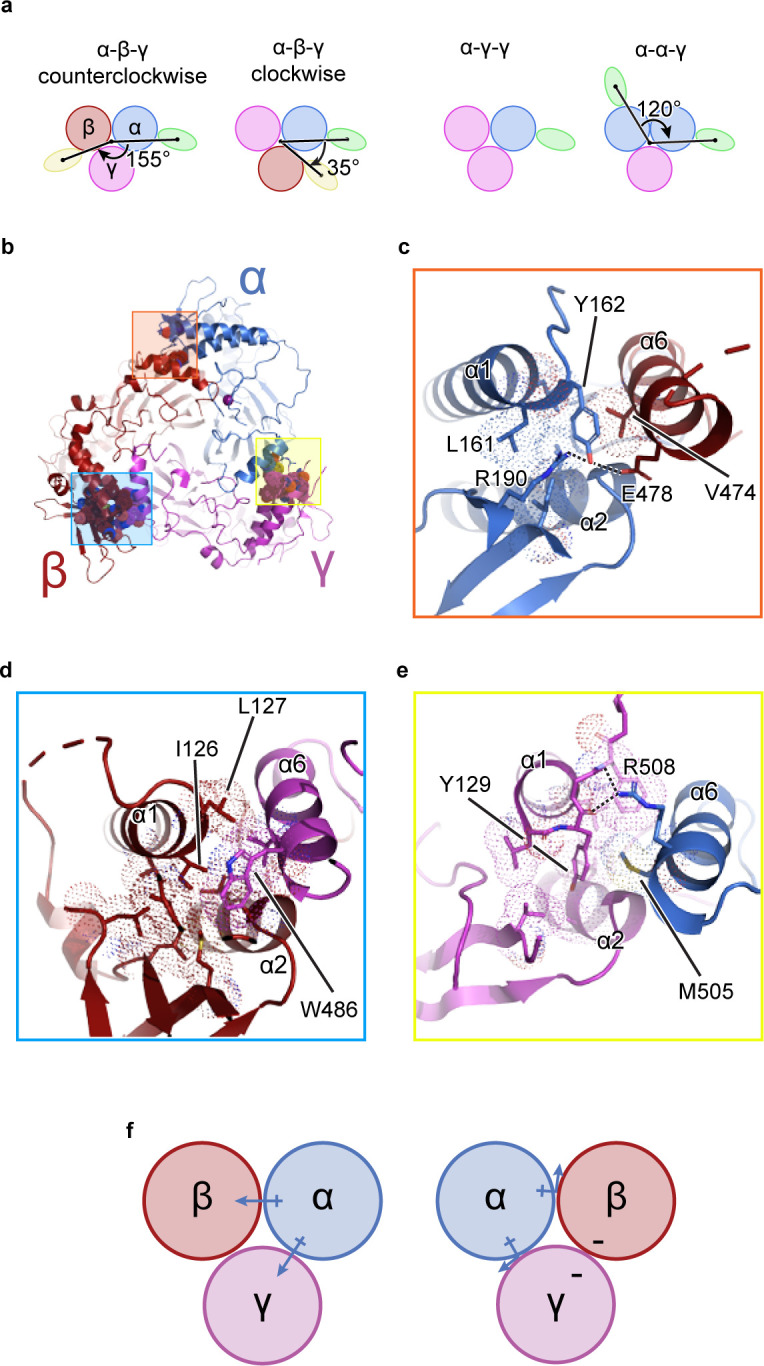
The unique molecular interactions at the subunit interface define heteromeric assembly of ENaC. (**a**) Top-down cartoon schematic illustration of ENaC with α-β-γ counterclockwise as resolved by cryo-EM (top left) and three possible assemblies of ENaC based on the defined purification scheme (see Materials and methods) as seen from left: α-β-γ clockwise (second panel), α-γ-γ (third panel), and α-α-γ (fourth panel). Subunits and Fabs are colored blue (α), red (β), magenta (γ), green (7B1) and yellow (10D4). (**b**) View of the ENaC_FL_ from the extracellular side and shown in cartoon representation. The α, β, and γ are colored blue, red, magenta, respectively. Boxed regions define subunit interactions near the top of the ECD. (**c**) Close-up view of the α-β interface as highlighted with an orange square in (**b**). The hydroxyl group of αTyr162 forms hydrogen bonds with αArg190 and βGlu478. Dashed lines indicate distances of 2.5–3.5 Å. (**d**) Zoomed-in view of the β-γ interface in blue boxed region. The equivalent residue βLeu127 is primarily interacting with residues in the adjacent α6. Instead, βIle126 resides in the equivalent position as in αTyr162 and γTyr129 makes van der Waals contacts with the residues from the α2, βGRIP, and the adjacent α6. (**e**) Enlarged view of the γ-α interface, yellow boxed region. The side chain of the equivalent γTyr129 is largely surrounded by hydrophobic residues. (**f**) Cartoon schematic illustration of the ENaC hydrogen bonding network. The α subunit donates hydrogen bonds to both the β and γ subunits in the counterclockwise arrangement (left). If the positions of α and β are swapped, the hydrogen bond donors and acceptors are mutually inaccessible (right).

It is known that functional ENaC channels require at least one α subunit (Canessa et al., 1994; Fyfe and Canessa, 1998; McNicholas and Canessa, 1997). Additionally, because the γ subunit gene contained the purification tag, all purified ENaCs contain at least one γ subunit (Figure 1—figure supplement 1a). Thus, if other combinations of ENaC heteromers were present, classes with one (α-γ-γ) or two Fabs (α-γ-β or α-α-γ) forming a 35° and 120° angle about the pseudo three-fold axis, respectively, would be observed (Figure 1a; Stewart et al., 2011; Baldin et al., 2020). However, no such classes were detected (Figure 1—figure supplements 2, 3 and 4a). To understand how ENaC favorably assembles as a heterotrimer with α-β-γ arranged counterclockwise, we inspected molecular interactions in the ECD at the subunit interface formed by the finger (α1 and α2 helices in all three subunits), the knuckle (α6 helix in all three subunits), and the GRIP domain (Figure 1). All subunit interfaces share van der Waals interactions between the first two helical turns of the α2 helix and the α6 helix of the adjacent subunit. Additionally, these α2 helices are capped by conserved serine residues (Figure 1—figure supplement 5).

**Figure 1—figure supplement 1.**
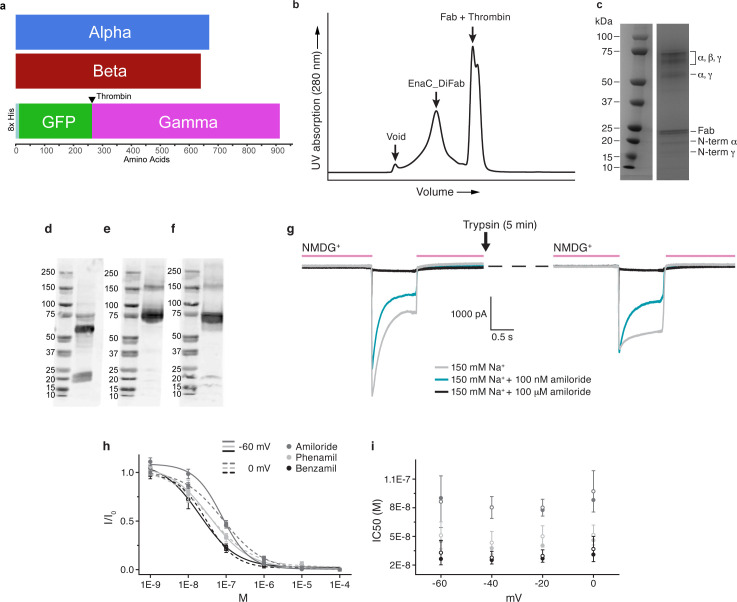
Biochemical and functional characterization of ENaC_FL_. (**a**) Schematic illustration of the ENaC_FL_ subunit constructs. (**b**) Size-exclusion chromatogram of purified ENaC_FL_ in complex with 7B1 and 10D4 Fabs (**c**) Representative SDS-PAGE of the purified protein. (**d**) Western blot (6 μg/ blot, rabbit polyclonal Ab to SCNN1A raised against amino acids 131–225, sc-21012) probing for the α subunit. The α lane has three bands, the uncut band at 75 kDa, and cut bands at approximately 60 and 20 kDa. This is expected due to the endogenous cleavage sites in WTα. (**e**) Western blot (2.5 μg/ blot, rabbit polyclonal Ab to SCNN1B raised against amino acids 406–640, ABclonal A1765) probing for the β subunit. As expected, majority of the band remains uncut at 75kD. (**f**) Western blot ( 6.25 μg/ blot, rabbit polyclonal Ab to SCNN1G raised against amino acids 100–250, ABclonal A15097) probing for the γ subunit. Majority of the band remains uncut at 75kD. Bands between 15-20kD are observed when γ is cut at the thrombin site at or near the thrombin and furin sites. (**g**) Representative current traces of ENaC_eGFP_ by whole-cell patch-clamp electrophysiology. Current amplitude mediated by ENaC_eGFP_ is sensitive to trypsin treatment based on increase of steady-state current. (**h**) Dose response curves of ENaC_eGFP_ at −60 mV and 0 mV for the three different pore blockers amiloride (grey), phenamil mesylate (light grey) and benzamil (dark grey). The dose response was determined using the following concentrations of each blocker, 1 nM, 10 nM, 100 nM, 1 μM, 10 μM and 100 μM. The amiloride-sensitive current response (Na^+^ current subtracted from Na^+^ current in presence of 100 μM blocker) was normalized to the amiloride-sensitive Na^+^ current with no blocker. Each dose response was repeated five times for each blocker, and a curve was fitted to mean ± S.E.M. (**i**) Plot of IC_50_ vs voltage shows that there is no voltage dependency. The IC_50_ was determined for each dose response and represent five independent experiments for each blocker (mean ± S.E.M). Closed circles represent the IC_50_ determined from a curve fitted to the average normalized current, open circles represents the mean value after fitting a dose response curve to each individual experiment and determining the IC_50_.

**Figure 1—figure supplement 2.**
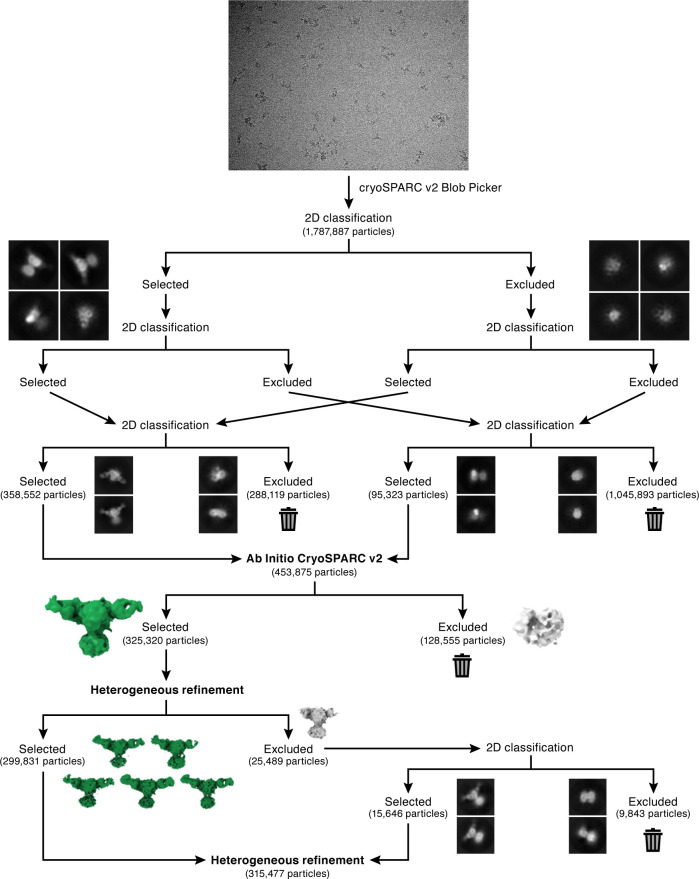
Cryo-EM initial data processing workflow. CryoSPARC Blob Picker was used for automated particle picking, to select an initial set of 1,787,887 particles. Multiple rounds of 2D classification were performed to select true-positive 2D classes of ENaC_FL_ as illustrated in the flowchart. After each round of classification, particles excluded from selection were combined and subject to 2D classification in order to recover true-positive 2D classes. After 2D classification, a final set of 453,875 particles were subject to 3D classification using cryoSPARC Ab Initio. Two classes were requested which resulted in one good class that contained 325,320 particles. Further 3D classification using cryoSPARC heterogeneous refinement was performed. Five of the six requested classes had similar features, and therefore combined. The sixth class was further classified by 2D classification to recover true-positive ENaC_FL_ classes, and added to a final stack of 315,477 particles.

**Figure 1—figure supplement 3.**
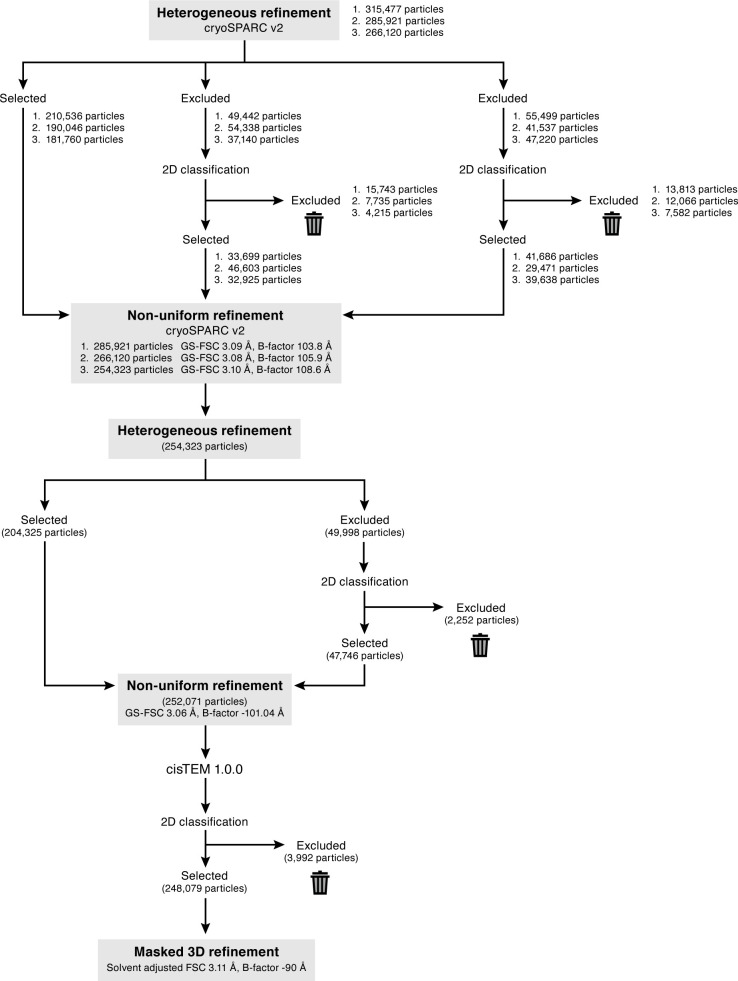
Cryo-EM data processing for the final map. To remove false-positive particles, three rounds of 3D classification was performed using cryoSPARC heterogeneous refinement. For each round, only the best class was selected, and the other two classes excluded. The excluded classes were classified by 2D classification to reveal true-positive ENaC classes, and then combined with the particles selected from the best class. The numbers listed (1-3) represent the number of particles in each round of refinement. Additionally, after each round of heterogenous refinement, the combined particle stack was refined using cryoSPARC non-uniform refinement. Three rounds of iterative heterogenous refinement and non-uniform refinement resulted in a stack of 254,323 particles. A final round of heterogeneous refinement resulted in a particle stack of 252,071 particles. Non-uniform refinement of this final set of particles resulted in a cryo-EM map of ENaC_FL_ at 3.06 Å resolution (gold-standard FSC), and a B-factor of ~ −100 Å^2^. This refined particle stack was also imported to cisTEM 1.0.0, classified by 2D classification, before final 3D refinement of 248,079 particles resulted in a cryo-EM map at a resolution of 3.11 Å (solvent-adjusted FSC).

**Figure 1—figure supplement 4.**
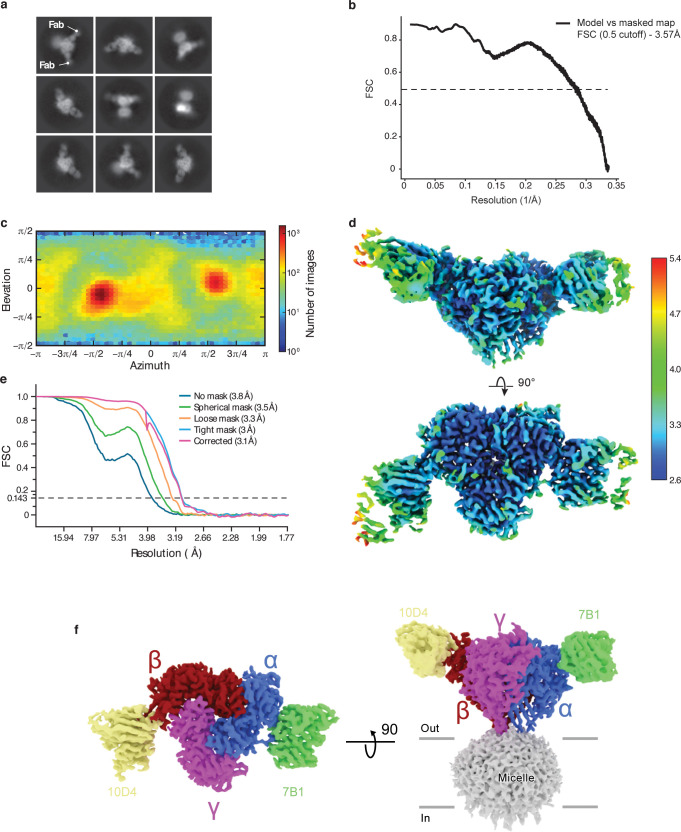
Cryo-EM analysis of ENaC_FL_ dataset. (**a**) Representative 2D class averages of ENaC_FL_. (**b**) Fourier Shell Correlation plot of model vs masked map. (**c**) Angular particle distribution. (**d**) Local resolution estimate (**e**) Gold-standard Fourier shell correlation (GS-FSC) (**f**) Final cryo-EM map of ENaC_FL_ with the α, β, γ, 7B1 Fab, and 10D4 Fab colored blue, red, magenta, green, and yellow, respectively.

**Figure 1—figure supplement 5.**
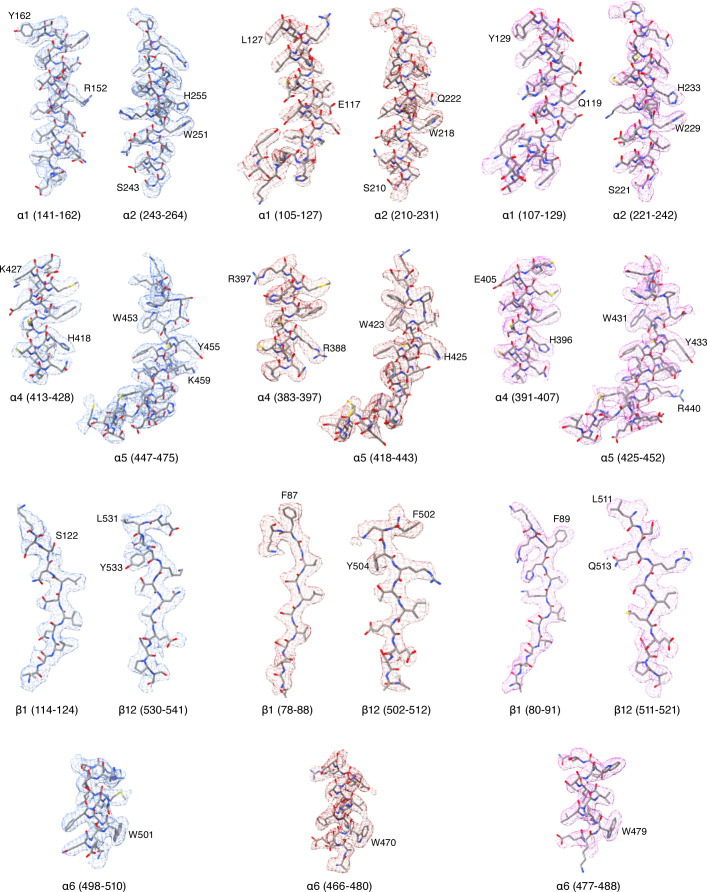
Cryo-EM potential maps of different regions in ENaC_FL_ map. The α, β, and γ subunit maps are represented in blue, red and magenta colored mesh, respectively. ENaC_FL_ coordinates are represented in grey stick superposed with the colored mesh maps.

**Figure 1—figure supplement 6.**
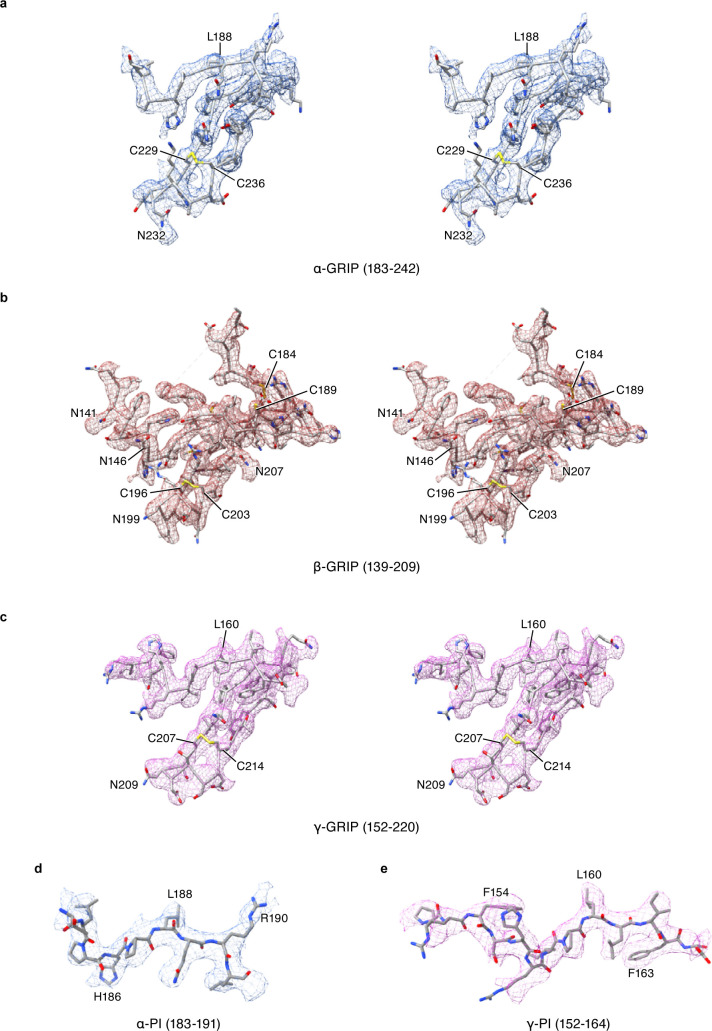
Stereoview of cryo-EM potential maps of the GRIP domain in ENaC_FL_ map. The α, β, and γ subunit maps are colored in blue, red and magenta colored mesh, respectively. The ENaC_FL_ coordinates are represented in grey stick superposed with the colored mesh maps.

By contrast, the interfaces formed by the α1 helix of one subunit and the α6 helix of the adjacent subunit show notable differences in both nonpolar and polar interactions. First, nonpolar contacts involve a tyrosine only found in α and γ; the equivalent residue is a leucine in β (βLeu127). The αTyr162 is surrounded by the hydrophobic αLeu161 and βVal474 (Figure 1c). The equivalent γTyr129, however, is tucked further into its own subunit, in a pocket comprising residues from the γ-α1 helix, γ-α2 helix, and γGRIP domain, as well as the adjacent αMet505 (Figure 1e). The nonpolar interactions at β/γ interface present yet another combination, in which two hydrophobic residues, βIle126 and βLeu127, make multiple hydrophobic contacts with the γ-α6 helix. In a conformation distinct to this interface, γTrp486 is wedged between the C-terminal end of the β-α1 helix and the βGRIP domain loop, locking the residue in place (Figure 1d). This conformation would result in a clash if the β and γ subunits were swapped, indicating that the positions of the aromatic residues may play a large role in defining the counterclockwise arrangement of channel subunits.

Second, polar interactions via hydrogen bonds are only found at two interfaces. The α/β interface αTyr162 is also poised to participate in a hydrogen bonding network with neighboring αArg190 in the αGRIP domain and βGlu478 (Figure 1c). Thus, the α subunit acts as a hydrogen bond donor to the β subunit (Figure 1f). However, at the γ/α interface, the γ subunit is a hydrogen bond acceptor, with the backbone carbonyl oxygens of γGly130 and γPhe131 forming hydrogen bonds with the guanidino group of αArg508 (Figure 1e). Finally, there is no clear hydrogen bond network at the β/γ subunit interface. Thus, the hydrogen bond networks at the different interfaces confer specificity for the counterclockwise α-β-γ channel (Figure 1f).

**Figure 2.**
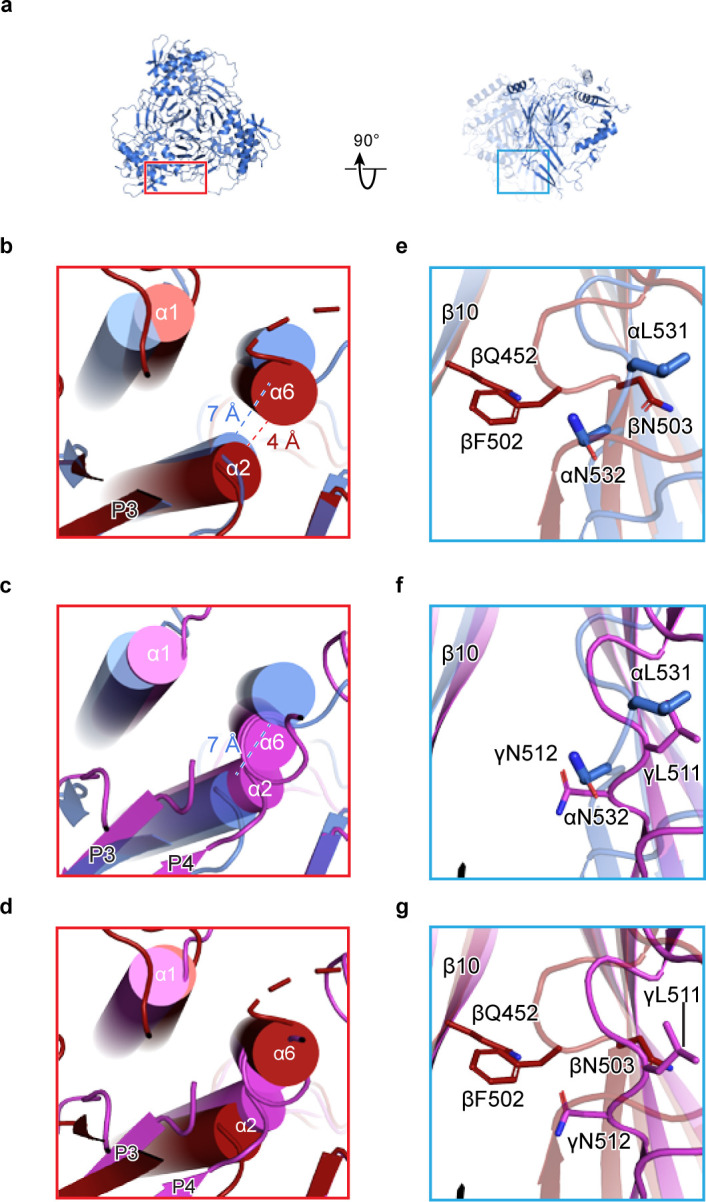
Human ENaC is a heteromeric channel with three different subunits. (**a**) Generated model of homomeric α ENaC using coordinates of the α subunit from the ENaC_FL_ structure. The two additional α subunits that complete the trimer were generated around the three-fold axis of symmetry. (**b–d**) Enlarged view of the subunit interface, from the red rectangular frame in (**a**), focusing on the α1 and α2 helices of the finger domain from one subunit and the α6 helix of the knuckle domain from the adjacent subunit. The homomeric trimers of α, β, and γ are superposed using the coordinates of the upper palm domain. Cartoon cylinders are colored as in (Figure 1). The α2 and α6 helices are spatially accommodated in the homomeric α (**b and c**) while minor and major clashes are observed in homomeric β (**b and d**) and γ (**c and d**), respectively. (**e–g**) Close-up view of the β10 strand from one subunit and the β11-β12 linker from the adjacent subunit. The observed conformation of the α (**e and f**) and γ (**f and g**) linkers is reminiscent of the β11-β12 linker conformations of the open and closed states of chicken acid-sensing ion channel 1. Conserved leucine and asparagine residues comprise the β11-β12 linker. The adopted linker conformation in β (**e and g**) is similar to that of the desensitized state of cASIC1. In this conformation, there is a steric clash between Gln452 of β10 and Phe502 of the β11-β12 linker.

We extended our analysis of homomeric channels by generating in silico models of homomeric forms of each ENaC subunit. To generate homomeric α, β, and γ channels (α_homo_, β_homo_, and γ_homo_), we used the coordinates of the ENaC_FL_ structure, assuming C3 symmetry around the three-fold axis (e.g. α_homo_, Figure 2a). We believe that this is a reasonable assumption, based on structures of the closely related ASIC. Comparison of these homomeric models reveals steric clashes in both the distal (finger/knuckle domain interface, Figure 2b–d) and core (lower palm/thumb domain interface, Figure 2e–g) ECD of the β and γ subunits. Focusing on the distal ECD, the β_homo_ channel α6 and α2 helices are 3 Å closer than the α_homo_ channel (Figure 2b), pointing to potential steric clash in the interface. The γ_homo_ channel appears even less stable in this region, with α6 and α2 clearly intersecting (Figure 2c). Conversely, in the core ECD, the β_homo_ channel shows clear steric clash between the β11-β12 linker and the adjacent β10 strand (Figure 2e). The core ECD of the γ- and α subunits are similar (Figure 2f) and, interestingly, the β11-β12 linkers are both similar to that of the ASIC open and closed states (Baconguis and Gouaux, 2012; Baconguis et al., 2014; Yoder et al., 2018). Meanwhile, the β subunit β11-β12 linker resembles that of ASIC trapped in the desensitized state (Gonzales et al., 2009). However, the functional consequences of the β11-β12 linker asymmetry, when comparing all three subunits, have not been investigated in detail, so caution is required in interpreting the state of the β11-β12 linker in the β subunit. None of these steric clashes are obvious in the α_homo_ channel, as expected given this channel’s ability to pass current in vitro.

**Figure 2—figure supplement 1.**
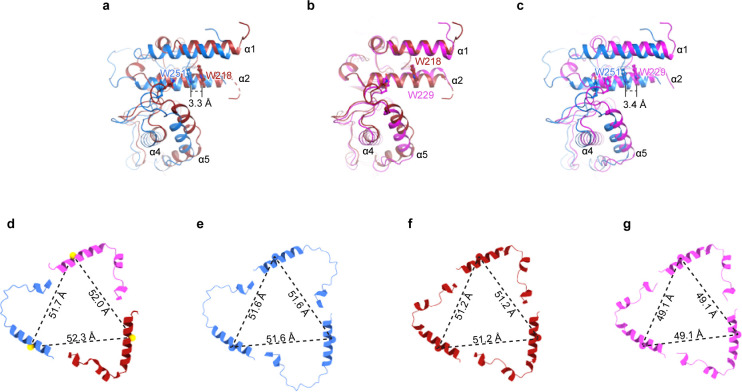
The domains within the ENaC subunits favor a heteromeric assembly. (**a-c**) Superpositions of the three subunits from the ENaC_FL_ structure at the upper palm domain show that the α subunit is distinct from the β and γ subunits. There is a rigid-body shift of the α finger and thumb domains relative to the equivalent domains in the β and γ subunits. Measured distances between the Cα atoms of the conserved Trp in the α2 helices show a shift of more than 3 Å in the finger domain region. (**d**) The cartoon representation of the α2 helices from α, β, and γ subunits. Cα atoms of the well-defined tryptophan residues in α, β, and γ (αTrp251, βTrp218, and γ229) are shown as spheres. They form a triangle with measured distances of ~52 Å. (**e–g**) Cartoon representation of the α2 helices of the generated homomeric models of α (**e**), β (**f**), and γ (**g**) show that the trimers form smaller triangles than the heteromeric form. The γ subunit makes the smallest triangles supporting the observed major clashes between the finger and knuckle domains, as shown in Figure 2.

We next determined how the domains within β and γ subunits arrange to give rise to steric clashes. To this end, we performed an alignment of the structure of each subunit by their highly similar upper palm domain. This alignment revealed a rigid-body shift of the finger (α1 and α2 helices) and thumb (α4 and α5 helices) domains in both β and γ subunits relative to the α subunit (Figure 2—figure supplement 1a–c). To determine the consequences of the shift in β and γ, we measured the distances between the Cα atoms of the conserved tryptophan residue in finger domain α2 helix in the homomeric models. The region is suitable for this analysis due to its greatly increased local resolution compared to the overall structure (Figure 1—figure supplements 4d and 5). Compared to ENaC_FL_, the distances between the Cα atoms of homomeric models, especially γ_homo_, are shorter (Figure 2—figure supplement 1d–g). As a result, the subunits are compressed toward the three-fold axis, causing major steric clashes.

### Identification of a putative Na^+^ binding site

**Figure 3.**
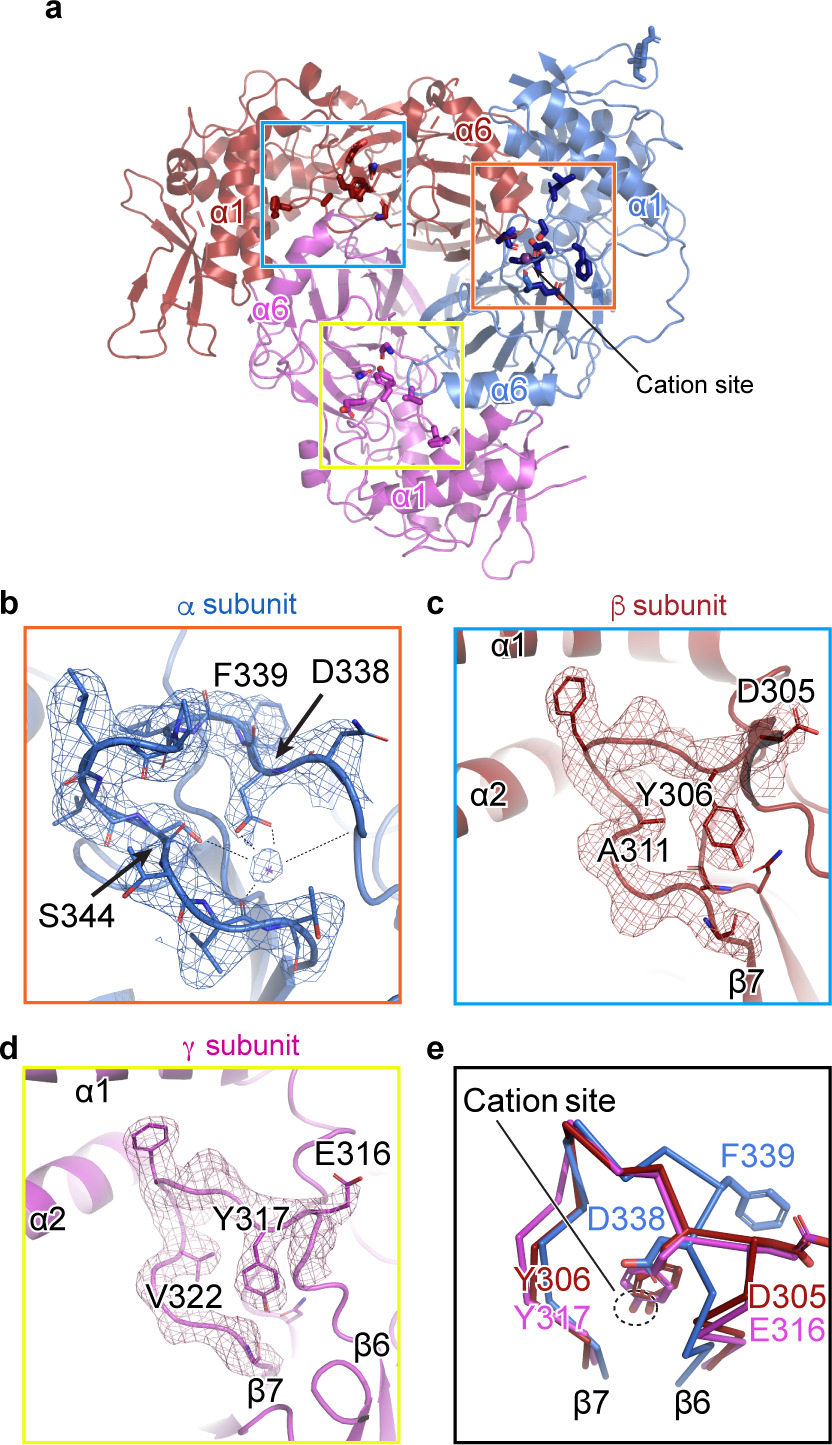
A cation binding site is located in the β6-β7 loop of the α subunit finger domain. (**a**) Cartoon representation of ENaC perpendicular to the membrane. α, β and γ are colored blue, red and magenta, respectively. The orange box shows the region of the cation site that is speculated to be a Na^+^ ion in the α subunit (**b**). The blue and yellow box represent the equivalent region, not occupied by a cation, in β (**c**) and γ (**d**) subunit, respectively. (**b–c**) Model of the β6-β7 loop superimposed with the potential map for the α subunit (**b**), β subunit (**c**) and γ subunit (**d**). (**b**) View of the cation site in the α subunit. The electrostatic potential of the β6-β7 loop and the Na^+^ is shown as a mesh. Dashed lines indicate distances of 2.5 – 3.4 Å. Residues shown with dashed lines are Leu135, Glu335, Asp338, and Val346. Ser344 is 3.8 Å away from the Na^+^. (**c, d**) Views of the equivalent regions in β (**c**) and γ (**d**). The residues occupying the equivalent position as Ser344 in α are alanine in β and valine in γ. (**e**) Superposition of the β6-β7 loops of all subunits demonstrate a striking difference in conformation in the α subunit. The acidic αAsp338 faces towards the Na^+^ site and Phe339 faces away from the cation site. The residues in the β and γ loops adopt a swapped conformation relative to the α subunit in which the aromatic residues are facing the equivalent sites while the acidic residues are exposed in solution.

We observed a map feature located near the β-ball domain and the β6-β7 loop of the α subunit, where residues αGlu335, αAsp338, and αSer344 in the α-β6-β7 loop have been identified as important in Na^+^ self-inhibition (Figure 3a,b; Kashlan et al., 2015). The map quality in this region is estimated to be well beyond 3 Å and thus the positions of the side chains are reliable. We speculate that this map feature is a cation, perhaps a Na^+^ ion, based on the surrounding residues, the above-mentioned functional studies, and the presence of high Na^+^ (150 mM) during purification, (Figure 3b). The cation interacts closely with several negative charges: the carboxyl group of αAsp338, and the carbonyl oxygens of αLeu135, αGlu335, and αVal346; all of these interactions are within distances of 2.5 – 3.5 Å. The hydroxyl group of αSer344 likely interacts with the cation via a water molecule, at a distance of approximately 4 Å. While these measured distances suggest that this feature is a positively charged ion, the cation site is perhaps not very highly selective for Na^+^. This is consistent with the ability of other cations like K^+^ and Li^+^ to reduce ENaC macroscopic currents, although the inhibitory effect of Na^+^ is larger in comparison (Kashlan et al., 2015; Bize and Horisberger, 2007). Indeed, definitive identification of this feature as the Na^+^ self-inhibition site would require resolving the structure of ENaC in the presence of K^+^ and determining if there are any associated structural changes.

We next examined the related positions in the β and γ subunits for a similar feature. The pocket into which Na^+^ binds in the α subunit is instead occupied by the side chains of βTyr306 and γTyr317 in their respective subunits (Figure 3c,d). Moreover, where αSer344 contributes a favorable polar interaction to the binding site, the equivalent positions in the β- and γ subunits are aliphatic (βAla311 and γVal322). In all three subunits, there is an acidic-aromatic residue pair at the segment of the β6-β7 loop believed to modulate Na^+^ self-inhibition. Superposition of this loop from all three subunits reveals that the α subunit adopts a swapped conformation relative to the β- and γ subunits near the putative cation binding site (Figure 3e). The acidic residues in the β- and γ subunits are exposed to solution, while the tyrosine hydroxyl groups are buried, participating in a network of internal hydrogen bonds. A phenylalanine in the equivalent position of the α subunit is incapable of participating in these hydrogen bonds and may explain the different conformation of the loop and, thus, the existence of the cation binding site.

### Characterization of the inhibitory peptide binding sites and GRIP domains

**Figure 4.**
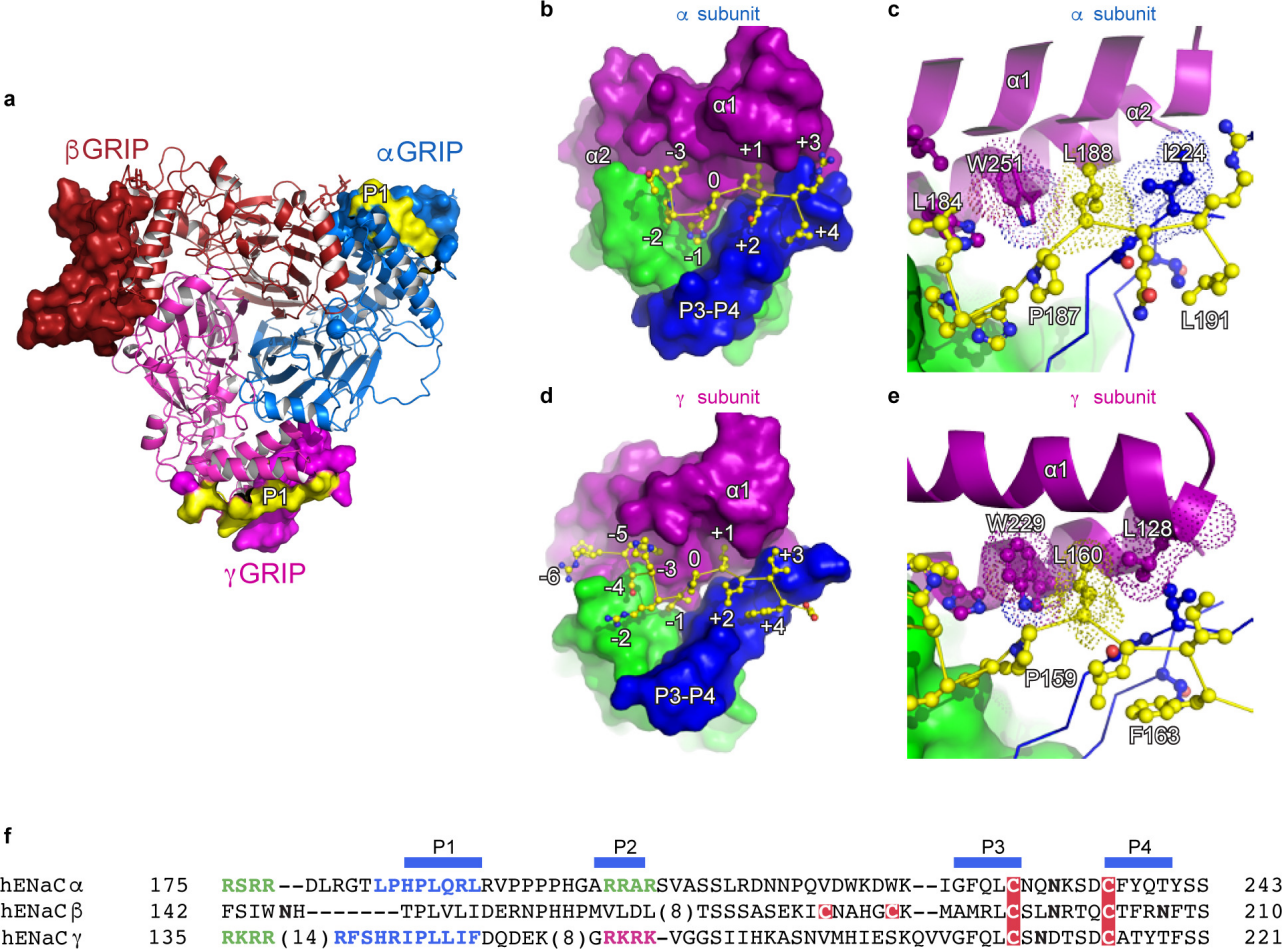
The inhibitory peptides in α and γ interact distinctly with the gating domains. (**a**) Cartoon representation of ENaC perpendicular to the membrane where the GRIP domains in all three subunits are shown as surface representation. α, β and γ are colored blue, red and magenta, respectively. Additionally, the inhibitory P1 peptides of αGRIP and γGRIP are highlighted in yellow. (**b**) Overall view of the inhibitory peptide pocket in the α subunit. The finger, thumb, and P3-P4 strands of the GRIP domains are colored purple, green, and blue, respectively, and shown in surface. The inhibitory peptide is shown in sticks representation and colored yellow. In the α subunit, the inhibitory peptide only extends to the *−3* position. (**c**) Close-up view of the pocket consisting of conserved residues Pro187, Leu188, and Trp251. Leu188 makes hydrophobic contacts with Trp251 from the α2 helix and Ile224 of the P3 strand of the GRIP domain. (**d**) Overall view of the inhibitory peptide pocket in the γ subunit. Representations are similar to (**b**). In the γ subunit, the inhibitory peptide extends to position *−6* making more extensive contacts with the finger and thumb domains. (**e**) Close-up view of the binding pocket consisting of the equivalent residues shown in (**c**). In the γ subunit, the Leu160 primarily interacts with the residues in the α2 helix. The residues in the GRIP domain that interact with the inhibitory peptides are in sticks representation in panels (**c**) and (**e**). (**f**) Sequence alignment of ENaC GRIP domains (hENaCα, GenBank ID:4506815; hENaCβ, 124301096; hENaCγ, 42476333). The sequences were aligned with Clustal Omega and manually adjusted. Coloring or shading is as follows: cysteines participating in disulfide bonds are in red boxes, glycosylation sites (predicted and/or present in cryo-EM map) are shown as bold N, furin sites are in green, prostasin site in magenta, and the P1 peptides in α and γ are in blue.

Proteolytic processing is one of the defining characteristics of ENaC gating, in which the removal of inhibitory P1 peptides, located in the α and γ subunit, shifts ENaC from a low to a high *P_o_* state (Carattino et al., 2008a; Passero et al., 2010; Figure 4). In the α subunit, the P1 peptide consists of residues α184–191 (LPHPLQRL) while the γ subunit P1 peptide includes γ153–163) RFSHRIPLLIF (Figure 4a,f). Because the residue numbers of the inhibitory P1 peptides vary across different species, we propose a numbering system in which the highly-conserved prolines (αPro187, βPro149, and γPro159) are denoted as position *0* to simplify discussion. Residues closer to the N-terminus are labeled as *-n*, while residues closer to the C-terminus are labeled as *+n*, for example αGln189, βVal151, and γLeu161 are each *+2* (Figure 4b,d). This naming convention echoes that of protease binding sites (Schechter and Berger, 1968). The N-termini of the P1 peptide are highly diverse in primary sequence, molecular organization, and interaction. The αP1 contains a histidine at *−1*, which inserts into a pocket formed by the thumb, finger, and P3 strand of the α subunit. A proline at *−2* changes the direction of the αP1 peptide, pointing the *−3* leucine toward the top of the α1 helix, which anchors the αP1 peptide between α1 and α2 helices (Figure 4b). The βP1 (N_-3_H_-2_T_-1_), on the other hand, forms a short, helical structure that is stabilized by a network of aromatic residues from both the α1 helix and the βGRIP domain (Figure 1—figure supplement 6). Finally, the γP1 (R_-6_F_-5_S_-4_H_-3_R_-2_I_-1_) binds a hydrophobic pocket in the thumb, finger, and P3 strand with its *−1* residue, just as in αP1. However, unlike αP1, γP1 has a solvent-exposed arginine at *−2* instead of a proline (Figure 4d and Figure 1—figure supplement 6e). Thus, the γP1 does not have the conformational constraint present in the αP1 that is introduced by a proline. Instead, we observe a clear map feature of γP1 that is extended alongside the finger domain in which the main chain and residues in γP1 forge multiple interactions with the thumb domain of the γ subunit (Noreng et al., 2018).

The C-terminal side of the P1 peptide primarily makes contact with the finger domain and the bulk of the GRIP domain. There is sequence divergence in the α subunit, with Q_+2_R_+3_L_+4_ as opposed to the hydrophobic sequences in the β (V_+2_L_+3_I_+4_) and γ (L_+2_I_+3_F_+4_) subunits. Additionally, each P1 peptide contains a conserved leucine residue at *+1* which forms hydrophobic contacts with a highly-conserved tryptophan from the α2 helix of the finger domain in all three subunits (αTrp251, βTrp218, and γTrp229, Figure 4c,e).

### Investigation of the GRIP domains

**Figure 5.**
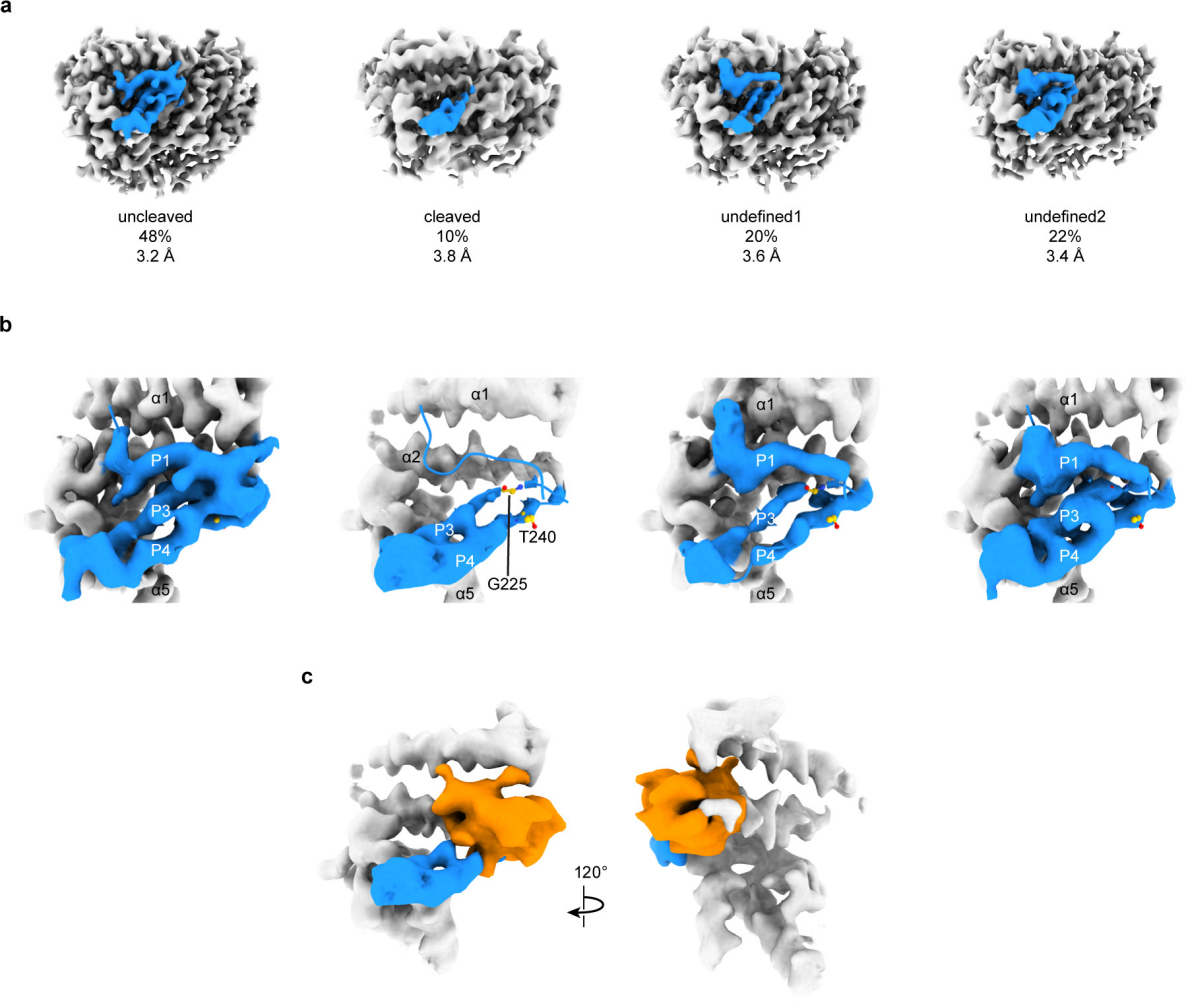
3D focused classification of the inhibitory peptide pocket in the α subunit reveals important site for ENaC regulation. (**a**) 3D classification of the GRIP domain in the α subunit revealed four major classes. Two classes clearly demonstrate the uncleaved and cleaved states of the α subunit representing 48% and 10% of the particles. Five classes were initially requested. The αGRIP domain is colored in blue. The remaining region of the ENaC map is colored gray for simplicity. (**b**) Close-up view of the GRIP domain. Compared to the uncleaved state, the map of the cleaved state shows that the region near the P3-P4/α2 is more disordered based on the lack of well-defined features that are observed in the uncleaved state. Residues that have been identified to markedly reduce peptide inhibition when mutated to tryptophans, Gly225 and Thr240, are shown in yellow and represented in sticks. (**c**) The difference map (orange) overlaps with the region in the GRIP domain that is absent or more disordered in the cleaved state.

The first structure of ENaC, referred to as ΔENaC which comprised of subunits with truncated amino and carboxy termini and other mutations in the ECD, demonstrated that all GRIP domains, including the protease-insensitive βGRIP, adopt similar anti-parallel β strand architecture (Noreng et al., 2018). The P3 and P4 strands of the GRIP domain (especially αGly225 and αThr240) have an outsize role in reduction of mouse ENaC current upon binding of the inhibitory peptide (Kashlan et al., 2010). In our ENaC_FL_ structure, the P3 and P4 strands are linked by a loop containing a predicted glycosylation site adjacent to the α5 helix of the thumb domain in all three subunits. Additionally, the important residue αGly225 is adjacent to αThr240 and forms hydrogen bonds with the C-terminal end of the αP1 peptide (Figure 5b; Blobner et al., 2018).

**Figure 5—figure supplement 1.**
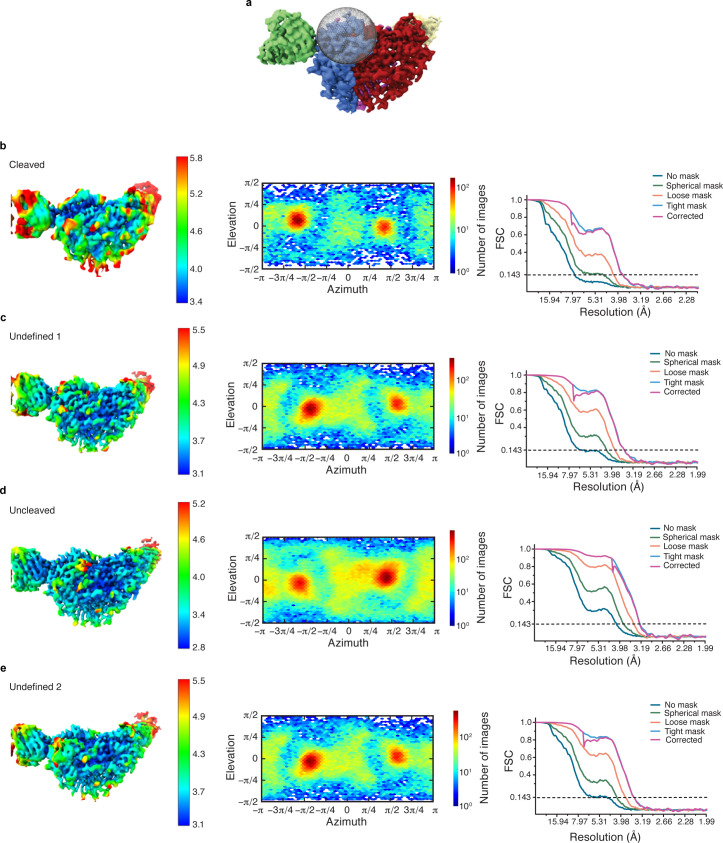
Cryo-EM data analysis of all α-GRIP classes in cryoSPARC. (**a**) Illustration of the region in which focused classification was performed. (**b–e**) Local resolution estimate, angular distribution and gold standard FSC of the resulting four maps from focused classification (as illustrated in Figure 5a) reveal that all classes have a similar overall particle distribution for the classification performed of the α-GRIP region.

**Figure 5—figure supplement 2.**
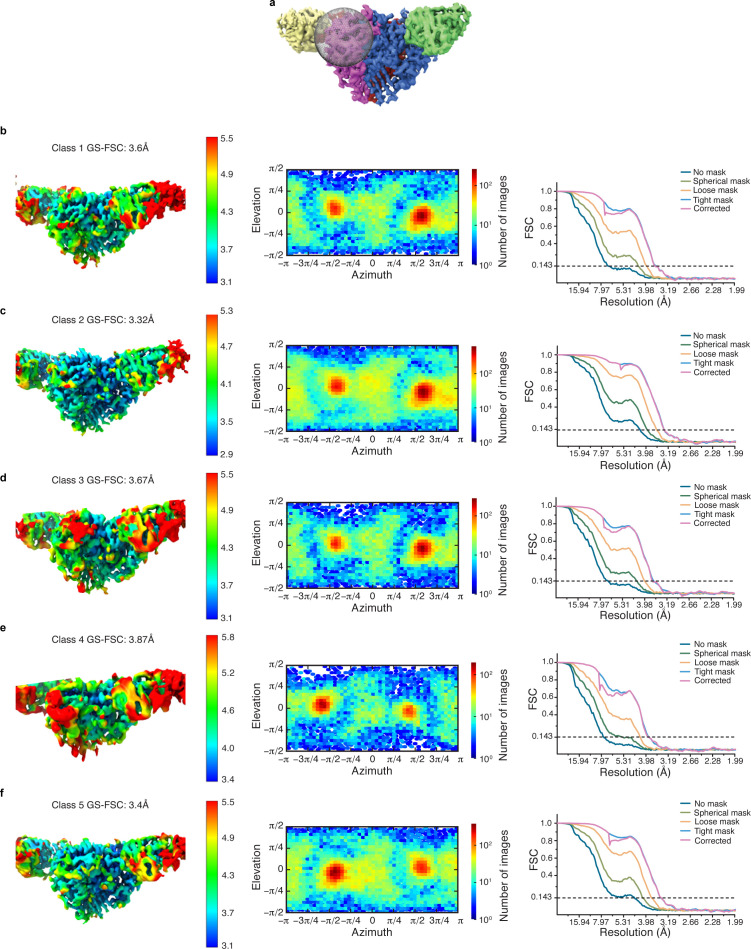
Cryo-EM data analysis of all γ-GRIP classes in cryoSPARC. (**a**) Illustration of the region in which focused classification was performed. (**b–f**) Local resolution estimate, angular distribution and gold standard FSC of the resulting four maps from focused classification reveal that all classes have similar overall particle distribution for the classification performed of the γ-GRIP region.

**Figure 5—figure supplement 3.**
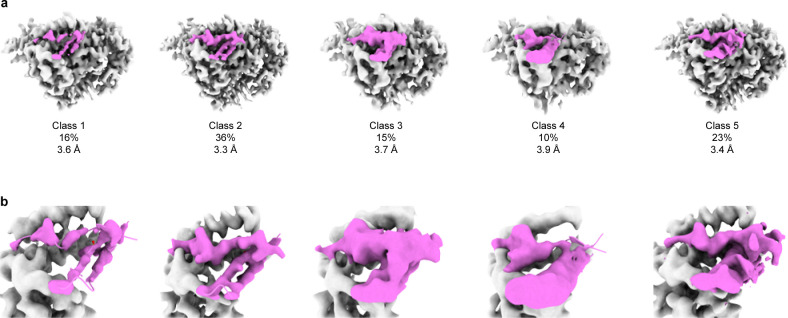
3D focused classification of the inhibitory peptide pocket in the γ subunit demonstrates heterogeneity in the inhibitory peptide cleavage states. (**a**) 3D classification of the GRIP domain in the γ subunit revealed five major classes. The major class representing 36% of the particles clearly demonstrate the uncleaved state of the γ subunit. As in the αGRIP classification, five classes were initially requested. The γGRIP domain is colored in magenta and the remaining region of the ENaC map is colored gray for simplicity. (**b**) Close-up view of the GRIP domain. Comparison of the GRIP domain maps reveals distinct features near the P3-P4 strands.

To further investigate the α and γ GRIP domains, we assayed the cleavage state of our sample by SDS-PAGE and western blot (Figure 1—figure supplement 1c–f). Our sample had partially proteolyzed α and γ subunits, as expected given the wild type GRIP domain. Using focused classification, we aimed to identify the different cleavage states - absence or presence of the inhibitory peptide – that our SDS-PAGE analysis suggests to exist. Particles from the final 3D refinement in both cryoSPARC and cisTEM were subjected to focused classification in cisTEM, centered on the αGRIP domain (Figure 5—figure supplement 1; Punjani et al., 2017; Grant et al., 2018). Assuming that the αGRIP domain is only cleaved at the canonical protease sites, there are four major combinatorial classes: uncleaved, fully cleaved, and two cleavage states at either protease sites. We requested five classes in each focused classification to allow for some heterogeneity in the particles.

In the αGRIP classification, the largest class (50% of the total number of particles) demonstrated features similar to ΔENaC, which could not be cleaved. We thus consider this the uncleaved class (Figure 5a,b and Figure 5—figure supplement 1d). We merged two classes which lacked features of the inhibitory peptide and the P3 strand into the fully-cleaved class (Figure 5a,b and Figure 5—figure supplement 1b). The fully-cleaved class contains 10% of the total particles. The other two classes, comprising 40% of the total particles, had a similar overall potential map to the uncleaved class, but contained some differences along the inhibitory peptide and P3 strand. We consider these classes undefined, and believe that they likely are an intermediate cleavage state or too low-contrast for proper classification (Figure 5a,b and Figure 5—figure supplement 1c,e). While on the one hand the western blot analysis showed a large population of cleaved α subunit, on the other hand, the focused classification analysis demonstrated a small population of the fully cleaved class. We speculate that there are three major reasons for the discrepancy. First, the existence of the relatively large class of undefined molecules in which the cleavage state of the P1 peptide is unresolved could contribute to the discrepancy between the observed intensity of the cleaved α band observed in western blot analysis (Figure 1—figure supplement 1d). Second, due to the binding site of our antibody spanning both cleavage sites, what appears to be a single large band may in fact represent both partially-cleaved and fully-cleaved α subunit (Noreng et al., 2018). And third, the set of particles used for focused classification was derived from rounds of 2D and 3D classifications, which removed denatured complexes and particles in thick ice, as examples. Thus, the population of particles used for SDS-PAGE and western blot analyses is not the same as the population used for focused classification. Nevertheless, implementing focused classification resulted in 3D maps that demonstrate differences in map features in the GRIP domain. A difference map between the cleaved and uncleaved maps shows a prominent feature overlapping the position of the uncleaved inhibitory strand, as expected (Figure 5c). The difference map also highlights deviations in the P3 strand potential, in agreement with observations in the cleaved and undefined classes indicating increased flexibility of this region upon cleavage. This disordered region begins near αGly225 in αP3 (Figure 5b). We thus speculate that αP3 becomes more mobile when the inhibitory peptide is proteolytically removed.

The γ subunit is known to be cleaved by several proteases aside from the canonical furin and prostasin, the latter of which is not present in our expression system (Kleyman et al., 2009). We expect these non-canonical cleavages, if present, to segregate into the undefined class. All five classes still showed features of the γ-inhibitory peptide (Figure 5—figure supplements 2 and 3). There are detectable differences in the inhibitory peptide between the five classes, with class one showing the most striking difference from the overall structure (Figure 5—figure supplement 3). We observed a small reduction of electron potential at the C-terminus of the inhibitory peptide and the γP3 strand, as expected. Nevertheless, this analysis suggests that the vast majority of the particles used for the initial map contain intact γGRIP domains.

### 7B1 Fab binds to the uncleaved and cleaved states of αGRIP

**Figure 6.**
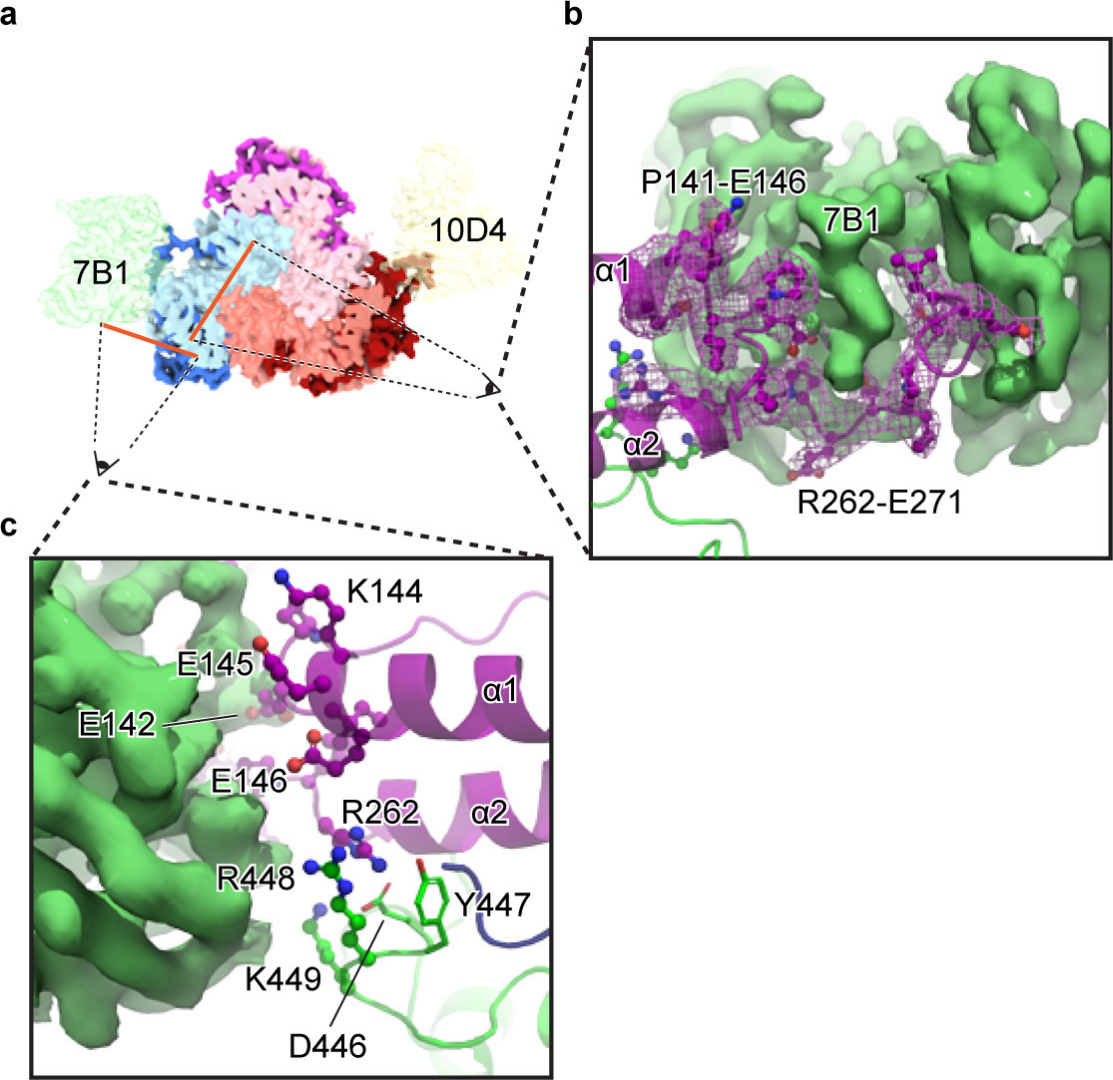
The 7B1 Fab binds to the α subunit making contacts with residues in the finger and thumb domains. (**a**) Top-down view of the ENaC_FL_ in complex with 7B1 and 10D4. The subunits and Fabs are colored as in Figure 1. (**b**) View of the interaction between the 7B1 Fab and the α2/α3 helices of the finger domain. The finger domain weaves within the binding region of the Fab. (**c**) Another view of the interaction between 7B1 and α subunit mediated by the finger and thumb domains. Acidic and basic residues that belong to the α subunit primarily mediate the interactions.

**Figure 7.**
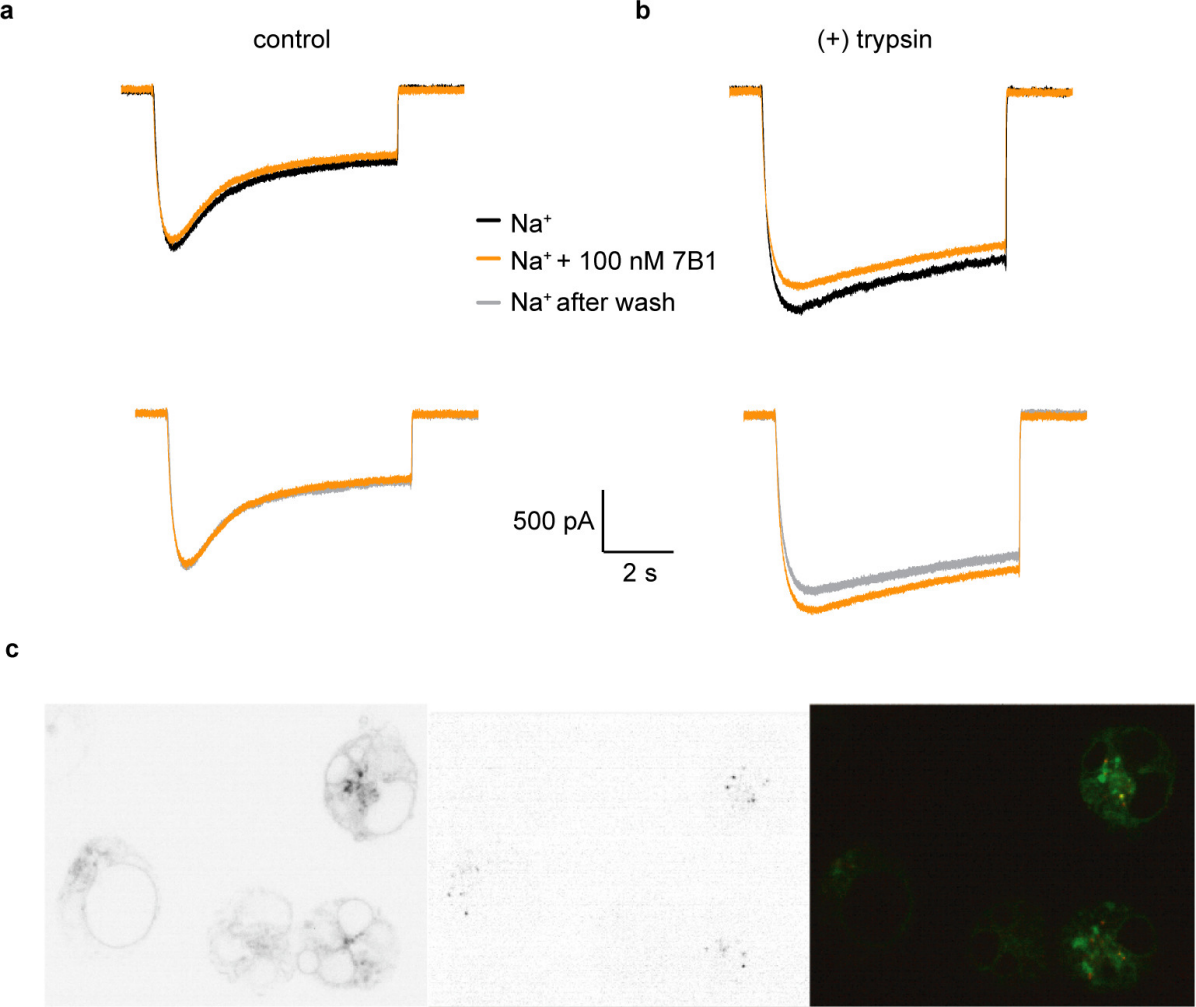
7B1 binds to the α subunit independent of the cleavage state of the α subunit. (**a**) Whole-cell patch clamp measurements of ENaC-mediated Na^+^ current indicate the 7B1 Fab does not alter current amplitude and shape. (**b**) Similar to the control current, 7B1 does not mediate acute effects of trypsin-cleaved ENaC. (**c**) Live confocal microscopy of HEK293S GNTI^-^ cells expressing ENaC_FL_ with eGFP fusion (left panel) are stained with TRITC labeled 7B1mAb (middle), recognizing the α subunit. The overlay of the GFP and TRITC channels (yellow, right panel) show that 7B1 mAb binds to ENaC_FL_ that are expressed on the cell surface. Images were acquired at a pixel size of 0.13 μm for two different wavelengths at 488 nm and 561 nm. The samples were binned 2 × 2 and the exposure time was 400 ms for 488 nm and 1 s for 561 nm.

Given that all of the classes (cleaved, uncleaved, and undefined of both α and γ subunits) have the same overall topology, we more closely investigated Fab binding and its effect on ENaC activity. 7B1 binds primarily to the finger domain and finger/thumb interface of the α subunit (Figure 6). 7B1 map feature at the αECD is equally strong in both the cleaved and uncleaved states of αGRIP and the cleaved and partially-cleaved states of γGRIP (Figure 5—figure supplements 1 and 2). Additionally, we did not observe any structural rearrangements in between the two states (Figure 5c), which does not align with the proposed gating mechanism derived from the structures of different functional states of ASIC (Yoder et al., 2018). It is possible that 7B1 traps ENaC in the conformation natively adopted by the uncleaved channel, regardless of the actual state of the channel. We thus assayed ENaC current before and after application of 100 nM 7B1 (10-fold greater than the observed K_D_ for purified ENaC_FL_, data not shown). If 7B1 traps the ECD in the uncleaved state, channel current would be low after application of protease in the presence of 7B1. We did not detect measurable acute differences in current magnitude or profile upon addition of 7B1 to either closed or open channels (Figure 7a,b). We confirmed surface binding by confocal microscopy of cells expressing ENaC_FL_ (Figure 7c). We can thus conclude that 7B1 binds ENaC at the cell surface, and that this binding does not reduce or modulate the macroscopic ENaC currents.

**Figure 7—figure supplement 1.**
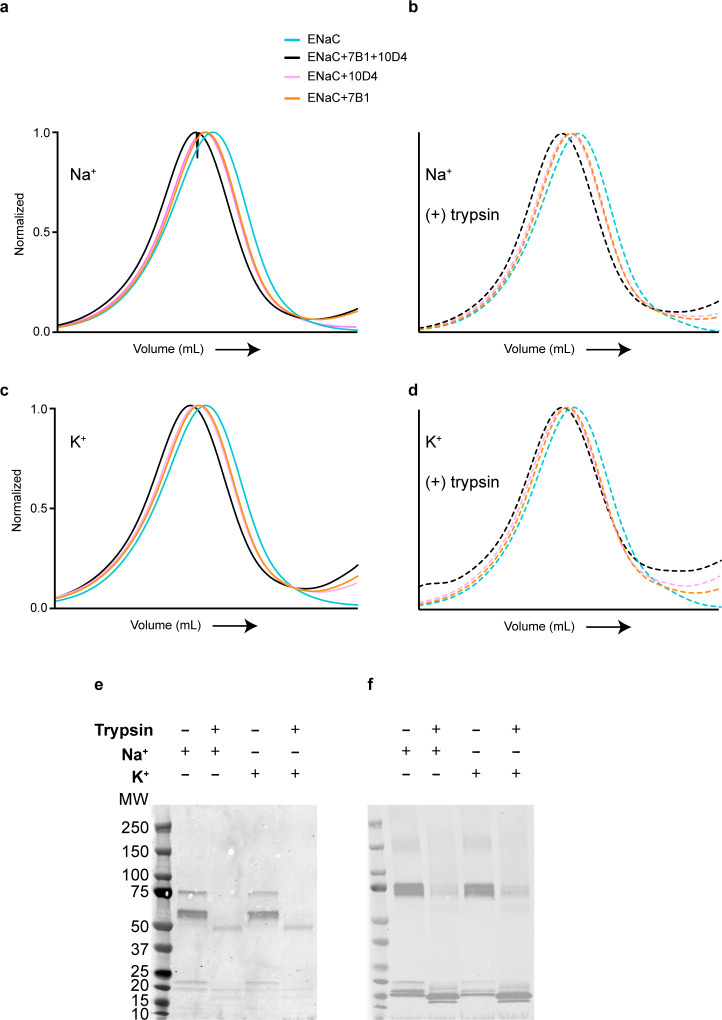
7B1 binds to both uncleaved and cleaved ENaC_FL_. (**a, b**) Biochemical characterization by Fluorescence size-exclusion chromatography (FSEC) shows that the 7B1 Fab binds to purified uncleaved (**a**) and cleaved ENaC_FL_ (**b**) in the presence of 150 mM NaCl and 20 mM HEPES pH 7.4. The cleaved ENaC_FL_ was exposed to trypsin prior to further clarification of the sample by size-exclusion chromatography. (**c, d**) The presence of K^+^ does not alter binding of 7B1 to uncleaved (**c**) and cleaved (**d**) ENaC_FL_ as determined by FSEC. (**e, f**) Western blot analysis of uncleaved and trypsin-cleaved ENaC_FL_ in the presence of 150 mM NaCl or 150 mM KCl. Samples were processed exactly as for FSEC. (**e**) The blot was probed with an antibody for the α subunit (αENaC, sc-21012). Before trypsin treatment, bands can be seen at 75 and 60 kDa which are expected sizes for α ENaC. Following trypsin treatment, those bands are no longer present and a new band is seen at approximately 50 kDa whether in a Na^+^ or K^+^ solution. (**f**) The blot was probed with an antibody for the γ subunit (γENaC, ab133430). Bands are seen at 75 kDa before addition of trypsin. Following trypsin treatment, there is decreased intensity of the 75 kDa band and new bands at smaller molecular weight indicating that the γ subunit is cleaved by trypsin. We do not observe a band near the 50 kDa MW marker likely due to loss of binding epitope for the antibody.

We also tested whether 7B1 binds only uncleaved ENaC. We performed fluorescence-detection size-exclusion chromatography (FSEC) and western blots of purified ENaC_FL_, either as-purified (mostly uncleaved) or treated with trypsin. Additionally, to assay whether 7B1 can bind any ENaC or only Na^+^-bound ENaC, we performed these experiments with an additional variable of Na^+^ or K^+^ buffer, the latter of which should not induce a Na^+^-bound conformation (Figure 7—figure supplement 1). As expected, uncleaved ENaC_FL_ binds both 7B1 and 10D4 (Figure 7—figure supplement 1). These results are in agreement with prior studies on ΔENaC. Purifying and analyzing the protein in K^+^ buffer showed no apparent difference in binding behavior. We thus conclude that 7B1 is able to bind uncleaved ENaC_FL_ in both high- and low-Na^+^ conditions. Encouragingly, this trend holds after ENaC_FL_ is treated with trypsin, moving the channels into a cleaved state (Figure 7—figure supplement 1). In summary, 7B1 can bind ENaC regardless of cleavage or Na^+^ concentration and does not modulate ENaC current. Thus, we believe that 7B1 does not trap ENaC in a closed-like conformation, and our classifications of the cleaved αGRIP structures are valid.

## Discussion

Here we employed single-particle cryo-EM to identify the structural determinants of subunit stoichiometry and arrangement in ENaC, and to illuminate the structural basis of ENaC modulation by Na^+^ and proteolysis. Functional analysis of different combinations of ENaC subunits demonstrated that robust Na^+^ currents were measured only when α, β, and γ were expressed together (Canessa et al., 1994). The first structure of human ENaC, ΔENaC, provided the first direct observation of the preferred assembly of the channel -- counterclockwise α-β-γ when viewed from outside the cell. Our new structure, ENaC_FL_, confirms the observed assembly and, with a more reliable placement of side chains, it deepens our understanding of the molecular principles that govern the heteromeric assembly of ENaC. The precise nature of how the subunits are positioned around the pseudo-three-fold axis involves an asymmetric arrangement of domains and unique molecular properties at interfaces.

The ECD of ENaC_FL_ deviates significantly from C3 symmetry. The swapped conformations of the β11-β12 linker in the β subunit and the β6-β7 aspartate and phenylalanine side chains in the α subunit are clear in the ENaC_FL_ map. The functional consequences of the swapped conformation of the β11-β12 linker in the β subunit are currently unknown but the findings provide a new direction for investigating the role of the β subunit in channel function. Furthermore, the presence of a phenylalanine adopting a different conformation in the α subunit from the tyrosines in the β and γ subunits, near the cation site confers a specialized function for the α subunit as the primary Na^+^ sensor.

Additionally, the high-resolution information of the GRIP domains in ENaC_FL_ allowed us to investigate the specific interactions between the inhibitory peptides and their binding pockets in the channel. We note that the loss of strong features in the P3 strands in the cleaved state is in agreement with functional studies of the α subunit by Kashlan and colleagues (Kashlan et al., 2010). While equivalent studies in the γ subunit have not been performed, our focused-classification maps of γGRIP indicate that similar structures and mechanisms exist between both the α and γ subunits. We speculate that the P3 strand anchors the N-terminal side of the α2 helix in place in the presence of the P1 peptide. After removal of P1, P3 is released, which destabilizes the α2 helix and β6/β7 interactions, disrupting the pocket for the novel α subunit Na^+^-binding site we identified in the present study. This provides a structural explanation for the observed loss of Na^+^ self-inhibition after proteolytic cleavage (Sheng et al., 2006). Surprisingly, we did not observe large-scale conformational differences in the α subunit between cleaved and uncleaved ECD maps. This is in contrast with the closed and open states of ASIC, in which the finger and thumb collapse in the open state. We confirmed that our tightly-binding 7B1 Fab does not trap the channel in a closed-like state. It is possible that the lack of observed conformational changes is a result of particle selection for an overall, closed structure, and then isolating those particles with a cleaved inhibitory peptide. It is possible that ENaC has a different gating mechanism from ASIC. Our maps highlight the importance of the α2 helix in mediating ENaC activity.

In this present study, we were unable to capture a fully cleaved state of γGRIP, which has a dominant effect on ENaC *P_O_* (Carattino et al., 2008b). The different classes derived from the focused classification analysis of the γGRIP all demonstrate that unlike the αP1 peptide, the γP1 peptide spans the finger domain forging extensive contacts with the thumb (Balchak et al., 2018). Whether complete removal of the γP1 peptide gives rise to conformational changes at the finger/thumb interface will be addressed by resolving a structure of a fully cleaved γGRIP. It is of vital importance to resolve the TMD and CD, especially the pore-forming region of ENaC. A recent structure of ASIC in a lipid bilayer reveals that a highly conserved ‘His-Gly’ (HG) motif forms a reentrant loop that lines the lower ion permeation pathway (Yoder and Gouaux, 2020). The HG motif is found in all ENaC/Degenerin family members and is critical for ENaC function (Gründer et al., 1997). With better methods for isolating ENaC with stable TMD and CD in addition to further improvement of sample preparation, we hope to resolve the full channel to understand the mechanistic link between removal of inhibitory peptides in the ECD and channel gating.

## Materials and methods

**Key resources table:** 

Reagent type (species) or resource	Designation	Source or reference	Identifiers	Additional information
Gene (*Homo sapiens*)	Amiloride-sensitive sodium channel subunit alpha isoform 1	Synthetic	NCBI Reference Sequence: NP_001029.1	Gene synthesized by BioBasic
Gene (*Homo sapiens*)	Amiloride-sensitive sodium channel subunit beta	Synthetic	NCBI Reference Sequence: NP_000327.2	Gene synthesized by BioBasic
Gene (*Homo sapiens*)	Amiloride-sensitive sodium channel subunit gamma	Synthetic	NCBI Reference Sequence: NP_001030.2	Gene synthesized by BioBasic
Cell line (*Homo sapiens*)	HEK293T/17	ATCC	Cat #ATCC CRL-11268	
Cell line (*Homo-sapiens*)	HEK293S GnTI-	ATCC	Cat #ATCC CRL-3022	
Antibody	7B1 mouse monoclonal	OHSU VGTI, Monoclonal Antibody Core	AB_2744525	Isotype IgG2a, kappa, 1:2 molar ratio
Antibody	10D4 mouse monoclonal	OHSU VGTI, Monoclonal Antibody Core	AB_2744526	Isotype IgG1, kappa. 1:2 molar ratio
Recombinant DNA reagent	pEG BacMam	Gift from Eric Gouaux	Doi: 10.1038/nprot.2014.173	
Chemical compound, drug	Amiloride hydrochloride hydrate	Sigma	Cat#: A7410	
Chemical compound, drug	Phenamil Mesylate	Tocris	Cat#: 3379	
Chemical compound, drug	Benzamil hydrochloride hydrate	Sigma	Cat#: B2417	
Other	TRITC	ThermoFischer	Cat#: 46112	
Software algorithm	HOTSPUR	Doi: 10.1017/s1431927619006792		
Software algorithm	MotionCor2	Doi:10.1038/nmeth.4193	SCR_016499	https://emcore.ucsf.edu/ucsf-motioncor2
Software algorithm	Ctffind4	Doi: 10.1016/j.jsb.2015.08.008	RRID:SCR_016732	https://grigoriefflab.umassmed.edu/ctffind4
Software algorithm	CryoSPARC	Doi:10.1038/nmeth.4169	SCR_016501	https://cryosparc.com/
Software algorithm	cisTEM1.0.0	Doi: 10.7554/eLife.35383	SCR_016502	https://cistem.org/
Software algorithm	pyem	Doi: 10.5281/zenodo.3576633		https://github.com/asarnow/pyem
Software algorithm	Pymol	Pymol Molecular Graphics System, Schrodinger, LLC	RRID:SCR_000305	http://www.pymol.org/
Software algorithm	UCSF Chimera	Doi: 10.1002/jcc.20084	RRID:SCR_004097	http://plato.cgl.ucsf.edu/chimera/
Software, algorithm	UCSF ChimeraX	Doi: 10.1002/pro.3235	RRID:SCR_015872	https://www.cgl.ucsf.edu/chimerax/
Software, algorithm	Coot	Doi: 10.1107/S0907444910007493	RRID:SCR_014222	https://www2.mrc-lmb.cam.ac.uk/personal/pemsley/coot/
Software, algorithm	Phenix	Doi:10.1107/S2059798318006551	RRID:SCR_014224	https://www.phenix-online.org/
Software, algorithm	MolProbity	Doi:10.1107/S0907444909042073	RRID:SCR_014226	http://molprobity.biochem.duke.edu

### Construct design

Two sets of constructs were designed for functional and structural studies. First, wild-type human α, β, and γ subunits were N-terminally fused with 8xHis tag, eGFP, and a thrombin recognition site (LVPRG); together, we refer to this set of constructs as ENaC_eGFP_. The ENaC_eGFP_ complex was ideal for whole-cell patch-clamp electrophysiology because the three eGFP per ENaC molecule facilitate in identifying ENaC-expressing cells. Second, for the biochemical aspects of the investigation, we put together another set of ENaC constructs in which the wild-type α and β subunits are untagged. As in ENaC_eGFP_, the wild-type γ subunit is N-terminally fused with an 8xHis tag, eGFP, and a thrombin site, and together with WTα and WTβ make a heteromeric ENaC_FL_. Because ENaC_FL_ only contains one eGFP per ENaC molecule, we reduced eGFP contamination during the purification step when using eGFP nanobody for affinity purification.

### Generation and isolation of Fabs

The protocol for generation and isolation of Fabs are as described in Noreng et al., 2018. Mouse monoclonal antibodies 7B1 and 10D4 were generated using standard procedure by Dan Cawley at the Vaccine and Gene Therapy Institute (OHSU). The 7B1 and 10D4 mAbs were previously selected because they recognize tertiary epitopes of ENaC. The mAbs were purified, and their Fabs were generated by papain cleavage. Fab 7B1 was isolated by anion exchange using HiTrap Q HP column while Fab 10D4 was eluted using Protein A column to remove Fc.

### Expression and purification of ENaC-Fab complexes

Human embryonic kidney cells (HEK293T/17) were grown in suspension at a density of 2 – 4 × 10^6^ cells/mL in Freestyle medium with 2% FBS and transduced with ENaC subunit virus to generate complexes ENaC_eGFP_ and ENaC_FL_ at a multiplicity of infection (MOI) of 1 and incubated at 37°C. 5 - 8 hr post transduction, amiloride was added to a final concentration of 1 μM, and cells were incubated at 30°C. After 24 – 48 hr, the cells were collected by centrifugation at 4790 xg for 20 min. The pellet was washed with 20 mM Tris, 200 mM NaCl and followed by a second round of centrifugation at 4790 xg for 15 min.

There were two approaches to purification of ENaC_FL_ that the cryo-EM data set arrived from. In both purifications, GFP-cleaved ENaC_FL_-diFab at pH 7.4 was the final purified complex used for cryo-EM sample preparation and will be referred to as ENaC_FL_. In one purification, membranes were prepared and ENaC_FL_ was purified from the prepared membranes, while in the second purification ENaC_FL_ was purified from the cell pellet.

In the first purification, cells expressing ENaC_FL_ were homogenized with a dounce homogenizer and sonicated in 20 mM Tris pH 7.4, 200 mM NaCl, 5 mM MgCl_2_, 25 U/mL nuclease and protease inhibitors. Lysed cells were centrifuged at 9715 xg for 20 min and the resulting supernatant containing the membrane fractions were further centrifuged at 100,000 xg for 1 hr. Membrane pellets were resuspended and solubilized in 20 mM Tris pH 7.4, 200 mM NaCl, 2 mM ATP, 2 mM MgCl_2, _1% digitonin (high purity, Millipore Sigma), 25 U/mL nuclease and protease inhibitors for 1 hr at 4°C. The solubilized fraction was isolated by ultracentrifugation 100,000 xg for 1 hr at 4°C.

In the second purification, cells expressing ENaC_FL_ were homogenized with a dounce homogenizer in 20 mM HEPES pH 7.4, 150 mM NaCl, 2 mM MgCl_2_, 25 U/mL nuclease and protease inhibitors. Homogenized cells were solubilized by adding the same buffer containing 2% digitonin (high purity, Millipore Sigma) and 4 mM ATP at 1 x initial volume (final volume 2x) for 2 hr at 4°C. The solubilized fraction was isolated by ultracentrifugation 100,000 xg for 1 hr at 4°C and supernatant was filtered through 0.45 μm filters.

Solubilized ENaC_FL_ (from both purifications) was bound to GFP nanobody resin by batch binding for 2 hr at 4°C. ENaC_FL_ bound to GFP nanobody resin was packed into an XK-16 column, and the column was washed with 20 mM Tris pH 7.4, 200 mM NaCl, 0.1% digitonin and 25 U/mL nuclease (second purification: 20 mM HEPES pH 7.4, 150 mM NaCl, 0.1% digitonin and 25 U/mL nuclease) followed by an additional wash of the same buffer containing 2 mM ATP. For elution, thrombin at 30 μg/mL and 5 mM CaCl_2_ in the same buffer was applied to the column and incubated for 30 min. GFP-cleaved ENaC_FL_ was eluted off with the same wash buffer and the eluted fractions were concentrated and then incubated with the Fabs 7B1 and 10D4 (DiFab complex) in a 1:2 molar ratio of ENaC_FL_:Fab for 10 min, and clarified by ultracentrifugation 100,000 xg for 1 hr at 4°C. The supernatant was injected onto a Superose 6 Increase 10/300 GL column equilibrated in 20 mM Tris pH 7.4, 200 mM NaCl, 0.1% digitonin (second purification: 20 mM HEPES pH 7.4, 150 mM NaCl, 0.1% digitonin) to isolate the protein complex by size-exclusion chromatography. Monodispersed peaks were pooled and concentrated to 2 – 3 mg/mL.

### Image acquisition and data processing

Purified GFP-cleaved ENaC_FL_-DiFab complexes at a concentration of 2 – 3 mg/mL was applied on holey-carbon cryo-EM grids which were glow discharged at 15mA for 60 s (Quantifoil Au 1.2/1.3 μm 300 mesh) prior to use. Grids were prepared using a Vitrobot Mark III (FEI) at 100% humidity and 12°C, where 3.5 μL of purified ENaC_FL_-DiFab complexes were applied followed by a manual blot on the side of the grid. Then another 3.5 μL of purified ENaC_FL_-DiFab complexes were applied before a wait time of 10 s, 3.5 s blot time at blot force 1, and then plunge frozen in liquid ethane cooled by liquid nitrogen.

Three large data sets were collected on the same microscope, a Titan Krios at the Multiscale Microscopy Core at OHSU, equipped with a Gatan K3 detector. One of the data sets, with a total of 9435 movies, were collected from the purification of ENaC_FL_ solubilized from membranes, while the other two data sets were collected from ENaC_FL_ purified directly from cells (see section ‘expression and purification’ for more details), one containing 9605 movies and the other containing 6153 movies. For all three data sets, movies were collected in super resolution mode with a pixel size of 0.415 Å. Total acquisition time was 3 s, and all three data sets were dose-fractionated to 60 frames with a dose rate of 1 e^-^/Å^2^/frame and total dose of 60 e^-^/Å^2^. Multishot with image shift between four holes was performed to speed up data collection using the automated acquisition program SerialEM (Mastronarde, 2003). Hotspur was used to initiate image alignment and ctf estimation during data collection (Elferich et al., 2019).

All data sets were binned 2 × 2 and motion corrected using motioncor2 (Zheng et al., 2017) with patch of 5 × 5. Each data set was processed individually using the software cryoSPARC v2 (Punjani et al., 2017) to determine the overall quality of final cryo-EM map before all three data sets were combined for final data processing. Defocus values were estimated using CTFFIND4 (Rohou and Grigorieff, 2015), and cryoSPARC blob picker was used for automated particle picking, initially resulting in 1,787,887 particles. Multiple rounds of 2D classification in cryoSPARC were performed where positive 2D classes of ENaC_FL_ were saved, and particles belonging to false-positive classes were combined and re-classified by 2D classification to further reveal and include true ENaC_FL_-diFab classes. After multiple rounds of 2D classification, a set of 453,875 particles was classified by cryoSPARCv2 *ab initio* and three rounds of 3D classification by heterogeneous refinement in cryoSPARCv2. To include as many true positive ENaC_FL_-diFab particles as possible, 3D classes of false – positive particles went through additional 2D classification and positive ENaC_FL_-diFab 2D classes were re-added for heterogeneous 3D classification.

The final data set containing 252,071 particles was processed using non-uniform refinement in cryoSPARC with default settings and C1 symmetry for a final 3D reconstruction with a Gold standard Fourier Shell Correlation (GS FSC) resolution of 3.06 Å. In addition, the same particles were exported from cryoSPARC by using the pyem conversion script (csparc2star.py) (Asarnow et al., 2019), and then imported to cisTEM 1.0.0 (Grant et al., 2018). In cisTEM particles were sorted by 2D classification, and 248,079 particles were refined with a mask that contained the ECD only to a solvent adjusted FSC of 3.11 Å. The final 3D map from 252,071 particles created in cryoSPARC v2 was used for model building and refinement. The improved resolution is potentially due to the advancement of the detector that was used for data collection (Gatan K2 switched to a Gatan K3 detector), as well as improvement of sample preparation where ENaC_FL_ grids were imaged in regions containing thinner ice.

To separate cleaved states of ENaC_FL_, focused classification (only refining the translational x and y parameters in cisTEM 1.0.0) was performed in the GRIP domain of the α and γ subunits. Subsequent classes obtained from focused classification were imported to cryoSPARC and *ab initio* was performed followed by non-uniform heterogeneous refinement to confirm the missing densities.

### Model building

The extracellular coordinates of the ΔENaC structure and the antigen-binding domains of 7B1 and 10D4 (PDB code: 6BQN [Noreng et al., 2018]) were docked into the cryo-EM map using Chimera (Pettersen et al., 2004). The coordinates were then manually inspected and adjusted using the computer program COOT (Emsley and Cowtan, 2004). The overall improved map quality shows many well-defined features that were not resolved in the ΔENaC map. These features include additional residues in the α- and γ-P1 peptides, Na^+^ ion, and N-acetylglucosamines (GlcNac). The final model contains all residues proposed to comprise the inhibitory peptides, LPHPLQRL and RFSHRIPLLIF, in the α- and γ-GRIP domains, respectively (Carattino et al., 2008a; Passero et al., 2010). Furthermore, seven glycosylation sites were modeled: two in α, four in β, and one in γ subunit.

Due to the lack of map features corresponding to the segments that connect the GRIP domains to the α1 and α2 helices, the loops were not included in the final model. Importantly, the ENaC_FL_ TMD was also excluded from the final model. While the 2D class averages and 3D maps demonstrate micelles features, which suggest the presence of the ENaC_FL_ TMD, the ion channel portion of the complex was not resolved. Overall, the following residues were modeled into the ENaC_FL_ cryo-EM map: residues 114–166, 183–191, 223–541 in α, 78–131, 139–167, 179–481, 486–512 in β, and 80–133, 152–164, 200–521 in γ. Iterative rounds of manual building and real-space refinement were conducted using COOT and PHENIX (Adams et al., 2011), respectively. The final model was determined to have good stereochemistry as assessed by MolProbity (Chen et al., 2010). Distance measurements and figures were made using the software Pymol (Schrodinger, LLC, 2015) and chimeraX (Goddard et al., 2018).

### Confocal fluorescence microscopy

Confocal fluorescence microscopy was performed as previously reported (Noreng et al., 2018). The antibody was conjugated to TRITC at a final dye:protein molar ratio of 3.7:1 in TBS.

### Western blotting

For western blots, ENaC_FL_ was purified as described above (solubilized with 20 mM DDM and 3 mM CHS instead of 1% digitonin). For the biochemical characterization of ENaC_FL_ as shown in Figure 1—figure supplement 1, the following polyclonal antibodies were used: sc-21012 (αENaC), ABclonal A1765 (βENaC), and ABclonal A15097 (γENaC). To validate purified ENaC_FL_ samples treated with trypsin, we also used western blotting as shown in Figure 7—figure supplement 1. The sample was split into groups, one kept untreated while the other was treated with 25 μg/mL of trypsin for 5 min at room temperature. Both samples were injected individually onto a Superose 6 Increase 10/300 column. The peak fractions from each condition were collected, pooled, and split into two groups. The first group was concentrated and prepared for FSEC and western blotting. The second group was concentrated and diluted multiple times with 0.5 mM DDM, 75 μM CHS, 20 mM HEPES pH 7.4, and 150 mM KCl to attain a NaCl concentration of approximately 0.24 mM and a KCl concentration of 149.76 mM. As a result, there were four total samples: uncleaved ENaC_FL_ in Na^+^ or K^+^ and cleaved ENaC_FL_ in Na^+^ or K^+^. SDS-PAGE samples of 2.9–3.2 μg each (the same amount within a blot) were loaded into the wells of 4 – 15% Tris-HCl Criterion Precast Gel. Proteins were electrophoresed at 180 V for 60 min and then blotted onto a nitrocellulose membrane at 80 V for 40 min. Membranes were blocked overnight at 4°C while rocking in 5% nonfat dry milk (NFDM). For staining, the primary antibody used was either αENaC (6 μg/ blot, rabbit polyclonal Ab to SCNN1A raised against amino acids 131–225, sc-21012) or γENaC (11 μg/blot, rabbit polyclonal Ab to SCNN1G raised against amino acids 100–200, ab133430). Primary antibodies were left on the membrane for 2 hr at room temperature while rocking. IRDye 680RD Goat anti-mouse IgG (LI-COR, 925–68070) was used as the secondary antibody on both blots. The secondary antibody was diluted to 1:25000 (1 μg/ 25 mL TBST) and allowed to bind for 1 hr at room temperature while rocking. The blots were imaged on an Odyssey western blot detection system.

### Whole cell patch clamp experiments

HEK293T/17 cells were grown in suspension at a density of 2 – 4 × 10^6^ cells/mL in Freestyle medium with 2% FBS and transduced with the virus (ENaC_eGFP_) at a multiplicity of infection (MOI) of 1 and incubated at 37°C. After approximately 5 hr the transduced suspension cells were incubated in the presence of 500 nM phenamil mesylate at 30°C for 12 – 14 hr. About 2–3 hr before recording, cells were transferred to wells containing glass coverslips at a density 0.3 – 0.5 × 10^6^ cells/mL and in Dulbecco's Modified Eagle Medium supplemented with 2% FBS and 500 nM phenamil mesylate. Whole cells recordings were carried out 17 – 24 hr after transduction. Pipettes were pulled and polished to 2.5 – 3.5 MΩ resistance and filled with internal solution containing (in mM): 150 KCl, 2 MgCl_2_, 5 EGTA, and 10 HEPES pH 7.4. For IC_50_ experiments, external solutions that were used contained (in mM): 150 NMDGCl or NaCl, 2 MgCl_2_ and CaCl_2_, and 10 HEPES pH 7.4. Increasing concentrations (1 nM, 10 nM, 100 nM, 1 μM, 10 μM, 100 μM) of amiloride, phenamil mesylate, or benzamil were added to the solution containing 150 mM NaCl. The macroscopic ENaC current was determined as the blocker-sensitive Na^+^-current that was blocked by 100 μM amiloride, phenamil mesylate, or benzamil. To determine the voltage sensitivity of each blocker, steps of +20 mV, from a starting holding potential at −60 mV up to 0 mV was performed for each experiment and the IC_50_ was determined for each voltage step (−60 mV, −40 mV, −20 mV and 0 mV). All recording experiments were repeated independently five times.

For whole cell patch clamp experiments to determine the effect of the monoclonal antibody (mAb) 7B1, current amplitudes were measured before and after addition of 100 nM of 7B1 for 3 min. Cells were then washed with buffer before application of trypsin (5 μg/mL) for 5 min to increase amiloride-sensitive Na^+^ currents. Post treatment with trypsin, cells were incubated with mAb 7B1 at a final concentration of 100 nM for 3 min. The amiloride-sensitive Na^+^-current was recorded before and after incubation with mAb.

## Data Availability

All cryo-EM maps have been deposited in the Electron Microscopy Data Bank under the accession code EMD-21896 for ENaC. Model coordinates have been deposited in the Protein Data Bank under the accession code 6WTH. The following datasets were generated: BaconguisI2020cryo-EM maps for ENacElectron Microscopy Data BankEMD-21896 BaconguisI2020Model coordinatesRCSB Protein Data Bank6WTH

## Additional files

Transparent reporting form
