# Supplementary material for: Molecular principles of assembly, activation, and inhibition in epithelial sodium channel
Source: eLife. 2020 Jul 30;9:e59038. doi: 10.7554/eLife.59038 (PMC7413742; doi:10.7554/eLife.59038)
Supplement: Transparent reporting form [file elife-59038-transrepform.pdf]

Westbrook Centre, Milton Road
Cambridge CB4 1YG
UK

P 01223 855340
W elifesciences.org
T @elife

eLife's transparent reporting form

We encourage authors to provide detailed information *within their submission* to facilitate the interpretation and replication of experiments. Authors can upload supporting documentation to indicate the use of appropriate reporting guidelines for health-related research (see EQUATOR Network), life science research (see the BioSharing Information Resource), or the ARRIVE guidelines for reporting work involving animal research. Where applicable, authors should refer to any relevant reporting standards documents in this form.

If you have any questions, please consult our Journal Policies and/or contact us: editorial@elifesciences.org.

Sample-size estimation

- You should state whether an appropriate sample size was computed when the study was being designed
- You should state the statistical method of sample size computation and any required assumptions
- If no explicit power analysis was used, you should describe how you decided what sample (replicate) size (number) to use

Please outline where this information can be found within the submission (e.g., sections or figure legends), or explain why this information doesn't apply to your submission:

Cryo-EM: Sample size of ~25,000 movies (three individual data sets) were collected for data processing to get to the final full-length ENaC map. The final resolution is based off of Gold standard FSC (0.143). The information of data processing and statistical analysis of the model built from the map is found in Figure 1 – figure supplement 1,2 and 3, and Figure 1 – table supplement 1. Description of sample preparation and imaging is found in the method section ("Expression and purification of ENaC-Fab complexes" and "Image acquisition and data processing").

Electrophysiology (whole cell patch clamp): All experiments were repeated independently five times for three different blockers (amiloride, phenamil mesylate and benzamil). ENaC currents were measured as amiloride-sensitive currents. To determine IC50, dose-response curves were normalized and a curve was fitted to the mean value +/- standard error of mean (S.E.M.). Description of electrophysiological data acquisition is found in Figure 1 – figure supplement 4, Figure 1 – table supplement 2, and in the method section "Whole cell patch clamp experiments"

Replicates

- You should report how often each experiment was performed

- You should include a definition of biological versus technical replication
- The data obtained should be provided and sufficient information should be provided to indicate the number of independent biological and/or technical replicates
- If you encountered any outliers, you should describe how these were handled
- Criteria for exclusion/inclusion of data should be clearly stated
- High-throughput sequence data should be uploaded before submission, with a private link for reviewers provided (these are available from both GEO and ArrayExpress)

Please outline where this information can be found within the submission (e.g., sections or figure legends), or explain why this information doesn't apply to your submission:

Cryo-EM: Multiple purifications and screening session were performed prior to the final data set in order to optimize imaging condition. Although majority of the cryo-EM maps were at a lower resolution (compared to final data set), all maps obtained from cryo-EM data collection and processing have revealed a similar cryo-EM map. The information of data processing and statistical analysis of the model built from the map is found in Figure 1 – figure supplement 1,2 and 3, and Figure 1 – table supplement 1. Description of sample preparation and imaging is found in the method section ("Expression and purification of ENaC-Fab complexes" and "Image acquisition and data processing").

Electrophysiology: All experiments were repeated independently five times. Outliers were cells that didn't form a gigaseal, or where the seal broke before the recording protocol was completed. Description of electrophysiological data acquisition is found in Figure 1 – figure supplement 4, Figure 1 – table supplement 2, and in the method section "Whole cell patch clamp experiments"

Statistical reporting

- Statistical analysis methods should be described and justified
- Raw data should be presented in figures whenever informative to do so (typically when N per group is less than 10)
- For each experiment, you should identify the statistical tests used, exact values of N, definitions of center, methods of multiple test correction, and dispersion and precision measures (e.g., mean, median, SD, SEM, confidence intervals; and, for the major substantive results, a measure of effect size (e.g., Pearson's r, Cohen's d)
- Report exact p-values wherever possible alongside the summary statistics and 95% confidence intervals. These should be reported for all key questions and not only when the p-value is less than 0.05.

Please outline where this information can be found within the submission (e.g., sections or figure legends), or explain why this information doesn't apply to your submission:

Cryo-EM: Statistical methods for map resolution was based off of gold standard FSC (0.143). Model refinement and validation was done using Phenix real space refine and MolProbity. Details are found in Figure 1 – figure supplement 3 and Figure 1 – table supplement 1.

Electrophysiology: All experiments were repeated five times. Statistical analysis involved fitting a dose response curve to normalized values to determine mean +/- standard error of mean (S.E.M.). Description of electrophysiological data acquisition is found in Figure 1 – figure supplement 4, Figure 1 – table supplement 2, and in the method section "Whole cell patch clamp experiments"

(For large datasets, or papers with a very large number of statistical tests, you may upload a single table file with tests, Ns, etc., with reference to sections in the manuscript.)

Group allocation

- Indicate how samples were allocated into experimental groups (in the case of clinical studies, please specify allocation to treatment method); if randomization was used, please also state if restricted randomization was applied
- Indicate if masking was used during group allocation, data collection and/or data analysis

Please outline where this information can be found within the submission (e.g., sections or figure legends), or explain why this information doesn't apply to your submission:

Cryo-EM: Gold standard FSC (0.143 cutoff) used for estimation of overall resolution. Data set was split in two(randomized) to measure the fourier shell correlation (FSC). Details found in Figure 1 – figure supplement 3 and method section "Image acquisition and data processing".

Electrophysiology: Healthy cells expressing GFP-tagged ENaC was randomly picked for ENaC current recordings. Method section "Whole cell patch clamp experiments" details the experimental setup.

Additional data files ("source data")

- We encourage you to upload relevant additional data files, such as numerical data that are represented as a graph in a figure, or as a summary table
- Where provided, these should be in the most useful format, and they can be uploaded as "Source data" files linked to a main figure or table
- Include model definition files including the full list of parameters used
- Include code used for data analysis (e.g., R, MatLab)
- Avoid stating that data files are "available upon request"

Please indicate the figures or tables for which source data files have been provided:

N/A
